# Zootherapy as a potential pathway for zoonotic spillover: a mixed-methods study of the use of animal products in medicinal and cultural practices in Nigeria

**DOI:** 10.1186/s42522-022-00060-3

**Published:** 2022-02-26

**Authors:** Sagan Friant, Jesse Bonwitt, Wilfred A. Ayambem, Nzube M. Ifebueme, Alobi O. Alobi, Oshama M. Otukpa, Andrew J. Bennett, Corrigan Shea, Jessica M. Rothman, Tony L. Goldberg, Jerry K. Jacka

**Affiliations:** 1 Department of Anthropology, The Pennsylvania State University, University Park, PA USA; 2 Huck Institutes of the Life Sciences, The Pennsylvania State University, University Park, PA USA; 3 Poxvirus and Rabies Branch, Division of High Consequence Pathogens and Pathology, Centers for Disease Control and Prevention, Atlanta, GA 30329 USA; 4 Department of Anthropology, Durham University, Durham, UK; 5 Department of Forestry and Wildlife Resources Management, University of Calabar, Calabar, Nigeria; 6 Department of Pathobiological Sciences, University of Wisconsin – Madison, Madison, WI USA; 7 Genomics and Bioinformatics Department, Biological Defense Research Directorate, Naval Medical Research Center–Frederick, Fort Detrick, Frederick, MD USA; 8 Department of Anthropology, Hunter College of the City University of New York, New York, NY USA; 9 Department of Anthropology, University of Colorado Boulder, Boulder, CO 80309 USA

**Keywords:** Zoonoses, Risk behavior, Human-animal interactions, Traditional medicine, Ethnomedicine, Ethnoepidemiology, Wildlife, One health

## Abstract

**Background:**

Understanding how and why people interact with animals is important for the prevention and control of zoonoses. To date, studies have primarily focused on the most visible forms of human-animal contact (e.g., hunting and consumption), thereby blinding One Health researchers and practitioners to the broader range of human-animal interactions that can serve as cryptic sources of zoonotic diseases. Zootherapy, the use of animal products for traditional medicine and cultural practices, is widespread and can generate opportunities for human exposure to zoonoses. Existing research examining zootherapies omits details necessary to adequately assess potential zoonotic risks.

**Methods:**

We used a mixed-methods approach, combining quantitative and qualitative data from questionnaires, key informant interviews, and field notes to examine the use of zootherapy in nine villages engaged in wildlife hunting, consumption, and trade in Cross River State, Nigeria. We analyzed medicinal and cultural practices involving animals from a zoonotic disease perspective, by including details of animal use that may generate pathways for zoonotic transmission. We also examined the sociodemographic, cultural, and environmental contexts of zootherapeutic practices that can further shape the nature and frequency of human-animal interactions.

**Results:**

Within our study population, people reported using 44 different animal species for zootherapeutic practices, including taxonomic groups considered to be “high risk” for zoonoses and threatened with extinction. Variation in use of animal parts, preparation norms, and administration practices generated a highly diverse set of zootherapeutic practices (*n* = 292) and potential zoonotic exposure risks. Use of zootherapy was patterned by demographic and environmental contexts, with zootherapy more commonly practiced by hunting households (OR = 2.47, *p* < 0.01), and prescriptions that were gender and age specific (e.g., maternal and pediatric care) or highly seasonal (e.g., associated with annual festivals and seasonal illnesses). Specific practices were informed by species availability and theories of healing (i.e., “like cures like” and sympathetic healing and magic) that further shaped the nature of human-animal interactions via zootherapy.

**Conclusions:**

Epidemiological investigations of zoonoses and public health interventions that aim to reduce zoonotic exposures should explicitly consider zootherapy as a potential pathway for disease transmission and consider the sociocultural and environmental contexts of their use in health messaging and interventions.

**Supplementary Information:**

The online version contains supplementary material available at 10.1186/s42522-022-00060-3.

## Background

Human interactions with animals are fundamental to the One Health approach, which recognizes the interdependence of human, animal, and environmental health. Human-animal interactions through animal husbandry, hunting, and butchering of wildlife are frequently-cited causes of zoonotic disease transmission and emergence in human populations [1, 2]. However, harvesting and preparing meat for consumption are among many activities that bring humans, wildlife, and their bodily fluids in close contact [3]. Zootherapy, the use of animals for traditional medicine and related cultural purposes (e.g., healing, witchcraft, rituals, charms, ceremonies, and festivals), involves medicinal and symbolic use of animals and animal by-products (e.g., excreta, fur, feather, bones, glands, etc.). Zootherapy may therefore be a source of zoonotic exposures. However, studies of zootherapies have predominantly analyzed these practices from ethnomedical, ethnozoological, and conservation perspectives, attending primarily to the species used and their medical purpose [4–12]. Minimal attention has been given to animal handling and the potential zoonotic risks associated with zootherapy [13–16]. Although cultural practices involving wild animals are well recognized as potential transmission routes for zoonoses [5, 13–15, 17, 18], efforts to understand the full extent of these practices have lagged behind more visible forms of human-animal contact, such as hunting and consumption.

The potential public health benefits of traditional medicine (usually therapeutic plants, but also minerals and animals) have been widely discussed [14, 19, 20]. Traditional medicine plays a role in meeting the healthcare needs of an estimated 70 to 95% of populations in low- and middle-income countries, especially those in Asia, Africa, Latin America, and the Middle East. In these countries, zootherapy offers a holistic approach to health and healing that is more accessible, acceptable, and affordable [21–25]. Comparatively little attention has been given to public health risks introduced from traditional medicine, specifically, zoonotic exposure risks from handling animals for medicine and other cultural practices [26]. While a majority of traditional medicine practices use plants, the use of animals (i.e. zootherapy) is also important and widespread [27, 28]. The increasing demand for traditional medicine contributes to unsustainable harvest and international trade in wildlife, which is a pressing conservation concern and threatens human health by allowing animals and their pathogens to travel across large distances [29, 30].

Rodents, bats, and primates consistently stand out as important wildlife reservoirs of zoonotic diseases [31–36], and these taxa feature prominently in zootherapy worldwide [4, 37, 38]. Rodents host the vast majority of mammal-borne zoonotic viruses [34, 39], and appear in zootherapies globally [8, 15, 40–43]. In a survey of 22 traditional medicine markets across Benin, rodents were the most abundant and speciose taxa represented, with nearly half of the reported local species found in traditional medicine markets [44]. Preparation and use of rodents as traditional medicine could provide less apparent opportunities for the transmission of rodent-borne zoonoses. For example, rodent-borne zoonoses transmissible to humans with poorly understood or unknown transmission routes, include: arenaviruses (e.g., Lassa virus, Lujo virus, and lymphocytic choriomeningitis virus), hantaviruses, orthopoxviruses (e.g., vaccinia, cowpox, and monkeypox viruses), and causative agents of bacterial diseases (e.g., plague, leptospirosis, rat bite fever, salmonella, and tularemia) [45]. Understanding the potential risks associated with zootherapeutic use of rodents and other animals requires an improved understanding of how potential hosts are handled, including details of the animal parts used, and their preparation and administration.

Bats host the highest number of zoonotic viruses per species, including: coronaviruses (e.g., MERS and SARS), lyssaviruses (e.g., rabies), filoviruses (e.g., Marburg viruses and potentially Ebola viruses), and henipaviruses (e.g. Nipah and Hendra viruses) [34]. Bat-associated zoonoses are of public health concern but the zoonotic origins and modes of transmission of many such diseases remain obscure [46–48]. For example, African fruit bats have been implicated as putative reservoirs of filoviruses responsible for viral hemorrhagic fever outbreaks [49–51], but documenting how spillover transmission occurs has proven challenging [52, 53]. Much of the attention surrounding human-bat interactions has focused on more visible forms of contact (e.g., hunting, butchering, and consumption) [54–60]. In addition to being hunted for food, bats are used in zootherapies globally, including the use of small house-dwelling bats (Nepal), bat fat (Pakistan), and bat teeth (Malaysia) for medicinal and cultural purposes [61–63]. Bats are also used to cure asthma, arthritis, and fever in Bangladesh [57], to enhance female fertility in Nigeria [64, 65], and are sold widely in traditional medicine markets in Benin [44]. The hunting and consumption of bats for meat and medicine involves at least 167 species in Africa, Asia, Oceania, and Central and South America, including reservoirs of zoonotic disease and threatened species [34, 38]. The widespread use of bats as zootherapy in areas where they are hunted and traded for bushmeat warrants deeper investigation into the potential role of zootherapy as a zoonotic risk factor [38].

Genetic similarity between humans and non-human primates may facilitate the transmission of a disproportionate number of zoonoses [66]. Primates are reservoirs of simian retroviruses that appear to regularly spillover into humans [67, 68], and in some cases evolved into epidemic or pandemic AIDS viruses [69]. Research and narrative surrounding the origins of AIDS is largely responsible for the current emphasis on bushmeat hunting as a major mode of zoonotic exposure [70–73]. Primates have also played roles in the transmission of Ebola virus [74, 75] and anthrax [76] to humans. In addition to being hunted for their meat, primates are widely used in zootherapies, with one global literature review indicating that 101 species of primates are used in traditional folk practices and magic–religious rituals, raising concerns for conservation of primates, of which 50% of species are in danger of becoming extinct [37]. Contact with non-human primates via zootherapy may be an unappreciated risk factor for exposure to primate pathogens globally.

Although rodents, bats, and primates occupy most of the thinking surrounding spillover transmission, there is a need to examine human contact with other taxa that serve as reservoirs or facilitate transmission to humans [32, 77, 78]. For example, wild ungulates have also been implicated in anthrax transmission [79] and are possibly involved in the natural ecology of Ebola virus [74, 80]. Contact with reptile body parts such as the skin, carapace, and blood can lead to the transmission of bacteria and helminths to humans [81]. Domestic animals can also play an important role in the ecology of wildlife pathogens, acting as amplification hosts that facilitate transmission to humans (e.g., Nipah and Hendra [82]). Indeed, domestic animals (primarily ungulates and carnivores) share a large number of infectious disease with humans [32, 78] and feature as both ingredients in and recipients of zootherapies globally [4, 13].

Deciphering potential zoonotic risks associated with zootherapy requires understanding human-animal interactions beyond simple tallies of species encounters and the purpose of their use. For example, zoonotic risks associated with zootherapy will depend on the animal product used, how it is prepared, and its route of administration. Zootherapeutic practices may be particularly risky when they involve direct contact with animals and their by-products (especially blood, urine, and feces), do not include heat inactivation during preparation (e.g., boiling, singing, smoking, grilling), or are administered subcutaneously (e.g., sharp injuries or splashes leading to exposure to broken skin or mucosa), topically (e.g., skin, eyes, ears, nose, vagina, rectum), orally, or via inhalation. To our knowledge there has been no previous examination of zootherapy from a zoonotic risk perspective, for instance, by including information on animal parts and details on associated preparation and administration that might affect exposure risks (see Additional File 1 for search strategy). In addition, attention to broader social, cultural, and ecological contexts of animal use is important for understanding the factors that influence potential public health risks and for designing effective and appropriate interventions for human health and biodiversity management [83–85].

Here, we characterized human-wildlife interactions shaped by zootherapy where hunting and zootherapy are core components of local cultures and livelihoods [15]. Our goal was to examine zootherapeutics across the diversity of animal taxa hunted, including details of the body parts used and how they were prepared and administered. This enables us to provide a comprehensive picture of the scope and scale of human-animal interactions via zootherapy and qualitatively assess exposure risks. We also examined the sociocultural and environmental factors that shape zootherapeutic use through mixed-methods analysis of the importance of different species and body parts, availability of animals used, and the sociodemographic and local factors that promote their use. Together, these data reveal potential risks associated with zootherapy and some of the sociocultural determinants of their use.

## Methods

### Study site

This study describes the cultural and medicinal uses of wildlife reported by communities within and near Cross River National Park in Southern Nigeria. The area is one of Africa’s most important biodiversity reserves, where, wild animals are both nutritionally and economically valuable [86], are important components of traditional medicine and cultural practices [15, 87], and are threatened from human activities [86, 88]. Cross River National Park is characteristic of lowland rainforest, forming a mosaic of disturbed and relatively undisturbed forest patches. The park contains the largest tract of remaining contiguous forest in Nigeria, high levels of species endemicity, and critically endangered (Cross River Gorilla [*Gorilla gorilla diehli*] and Pruess’s red colobus monkey [*Procolobus preussi*]) and endangered (Nigeria-Cameroon chimpanzee [*Pan troglodytes ellioti*], Drill monkey [*Mandrillus leucophaeus*], and Preuss’s monkey [*Allochrocebus preussi*]) species [89–92].

Social organization across our study area is based on politically decentralized villages with several clans under the leadership of chiefs. People are traditionally polytheistic, with beliefs and folklore centered on Sky, Earth, and Water Gods and a forest environment filled with supernatural beings [24]. Human-animal relationships are featured in nearly every aspect of cultural history in the region; including religious beliefs, folklore, art, secret societies, juju (i.e., charms and spells), gender identities, birth customs, initiations, and funerals [93]. Throughout the region, people engage in a mixed economy of hunting, gathering, and farming, subsisting off cultivated staple foods (e.g., cassava, yam, maize), as well as wild and cultivated fruits, vegetables, and animals. Hunting for subsistence, zootherapeutic and cultural uses, and trade threatens biodiversity and human health in the park and surrounding areas [15, 94]. In addition to treating and preventing health challenges, zootherapy in Nigeria also accommodates situations that are psychological, spiritual, or even mystical [7].

Our study involved nine villages representing four ethnic groups (Ejagham [*n* = 3 communities], Boki [*n* = 2], Ayo [n = 3], and Idoma [*n* = 1]) (Additional File 2). Villages were categorized by their location relative to the national park (i.e., within the park, within park support zone, outside of the park). Study communities were accessible by vehicle, motorbike, boat, and/or by foot only, and had variable access to government healthcare facilities. Two of the villages were designated as “enclaves” within Cross River National Park and had no formal health care workers or active medical facilities during our study period. Villages in the support zone had more active health care centers with variable presence of healthcare workers and resources. No rural health facilities had diagnostic capacity for zoonotic diseases.

The study team has extensive research and lived experience within the region, which helped us to cultivate relationships of confidence within the communities. SF has worked within Cross River National Park communities for 15 years and WAA, AOA, NMI, and OMO are from southern Nigeria. All study team members that were engaged in data collection lived within each village for a minimum of one month during data collection, where we participated in community work events, hunting excursions, community meetings, and festivals. Together, these shared experiences helped to enhance our awareness of the sensitivity of topics, further build confidence with respondents, and aided our analyses and interpretation of our results.

### Data collection and analysis

We used a mixed-methods approach [95], combining elements of quantitative and qualitative data and analyses from interviews and field notes. We collected data over two periods (2012 and 2017) as part of two larger studies of human-animal interactions focused on hunting [15] and consumption [86, 96] of wild animals. Here, we compiled data on zootherapeutic practices to investigate broader patterns and contexts of human-animal interactions in communities that hunt and consume wildlife. We did not compare our results from each study due to differences in sampling strategies, study sample sizes, and interview methodologies (Additional File 2). In 2012, we administered questionnaires orally to hunters who were purposively selected and then matched with randomly selected non-hunters in five communities (*n* = 327 males); however, because participants who did not identify as a hunter continuously revealed engagement in hunting activities, the resulting sample had a disproportionate number of hunters (*n* = 188, 57%) compared to non-hunters (*n* = 139, 43%) [15]. During individual interviews, we used species lists and published drawings to elicit zootherapeutic uses of wildlife [97]. Following a series of multiple-pass questions, where we used drawings and local names of wild animals to elicit information on hunting and consumption practices, we asked people if any of the animals shown, or any other animal, was used for medicinal purposes. If a respondent answered “yes”, we asked them to identify the species and body part, describe the methods of preparation and administration, and give the purpose of its use. We then asked participants if they had ever personally used any of the listed medicinal uses for themselves or their dependents. We then repeated the same process, asking participants to identify other uses of wild animals for cultural purposes, providing them with the examples of festival/ceremony, sacrifice, or charm. While we did not ask specifically about the use of fish, invertebrates, or human parts or by-products, which have been documented in zootherapy studies globally [11, 43, 98], we included these data if they were spontaneously mentioned. We collected additional sociodemographic information on age (*years*), household size (*number*), engagement in hunting or trapping animals in previous year (*y/n*), education level (*primary school/ beyond primary school*), wealth (*0–16*), and awareness of zoonoses (*y/n*) (see [15] for additional details). We used logistic regression to examine the effect of age, household size, engagement in hunting, education, wealth, zoonotic disease awareness, and village location on individual use of animals for medicine. We used backward elimination of predictor variables, initially including all variables in the model, but retaining only significant variables (α < 0.05) and first order interactions among significant main effects in the final model. We performed analyses with the *glm* function in R (4.0).

In 2017, we collected additional data on the cultural salience (i.e., importance/ cognitive accessibility) of different animals used for zootherapy. Specifically, we used free-listing and ranking exercises with key informants (*n* = 50 [average = 8 per village], representing 34 men and 16 women), recruited to include people with specialist knowledge of wild animals (e.g., hunters and traders, restaurant owners, traditional healers). We asked participants to free-list and then rank representative images of animals (1 = most important) across different domains, including medicinal and cultural practices. We asked other questions first, including the importance of animals for hunting, income, and taste preferences, which allowed participants to warm up into more sensitive questions about medicinal and cultural practices (published elsewhere [86, 96]). We then elicited further details on zootherapeutic uses via follow-up questions during free-listing exercises, including part of the body, purpose of its use, and details on preparation and administration. To determine the importance of different animals used for zootherapy, we constructed salience plots using a salience index (Smith’s S) combining the average rank assigned to each animal and the frequency it was mentioned as useful for medical uses or cultural purposes [99]. Freelists were analyzed in R statistical software using the *AnthroTools* package [100].

$$S=\sum \frac{inverted\ item\ \mathit{\operatorname{rank}}/\# items}{\# informants}$$

We organized descriptions of medicinal and other cultural uses from 2012 and 2017 into tables indicating the animal used, the number of times the animal was mentioned, the body part used, the purpose of its use, and details of preparation and administration practices. Because medicinal and cultural practices could not always be clearly differentiated (e.g., use of animals to enhance intelligence), we used the respondent’s categorization to guide our analysis. Zootherapies were considered distinct if they involved different species, parts of the body, were used for different and unrelated purposes, or varied in their preparation (e.g., use of raw vs. cooked parts) or administration (e.g., oral vs. topical administration). Treatments involving different body parts, but applied together, were treated as a single zootherapy. Several taxa could not be reliably identified due to low taxonomic certainty by informants. These taxa were grouped in our analyses at the most specific taxonomic group possible: monkeys, bats, pottos, galagos, pangolins, and fish.

## Results

### Zootherapies

We recorded a total of 292 zootherapeutic uses, including 172 medicinal uses and 120 other cultural uses of animals (Additional File 3). Animals were used as traditional medicine for purposes including treatment for various injuries and ailments from burns to epilepsy, behavioral problems (e.g., nighttime incontinence), mental problems, and as a cure for poisoning. Animal parts were also used as vessels for medicine (e.g., skulls and shells to make, hold, and deliver medicine) and as health promoters (e.g., bones to confer strength). Other cultural practices included the use of animals for: ceremonial consumption (e.g., for festivals or major life events), sacrifice, charms/juju (e.g., spells used in religious practice, for protection, or good fortune), display (e.g., decoration or prop), gifts, payment of fines, tools, and as a poison.

### Animal use

**Fig. 1:**
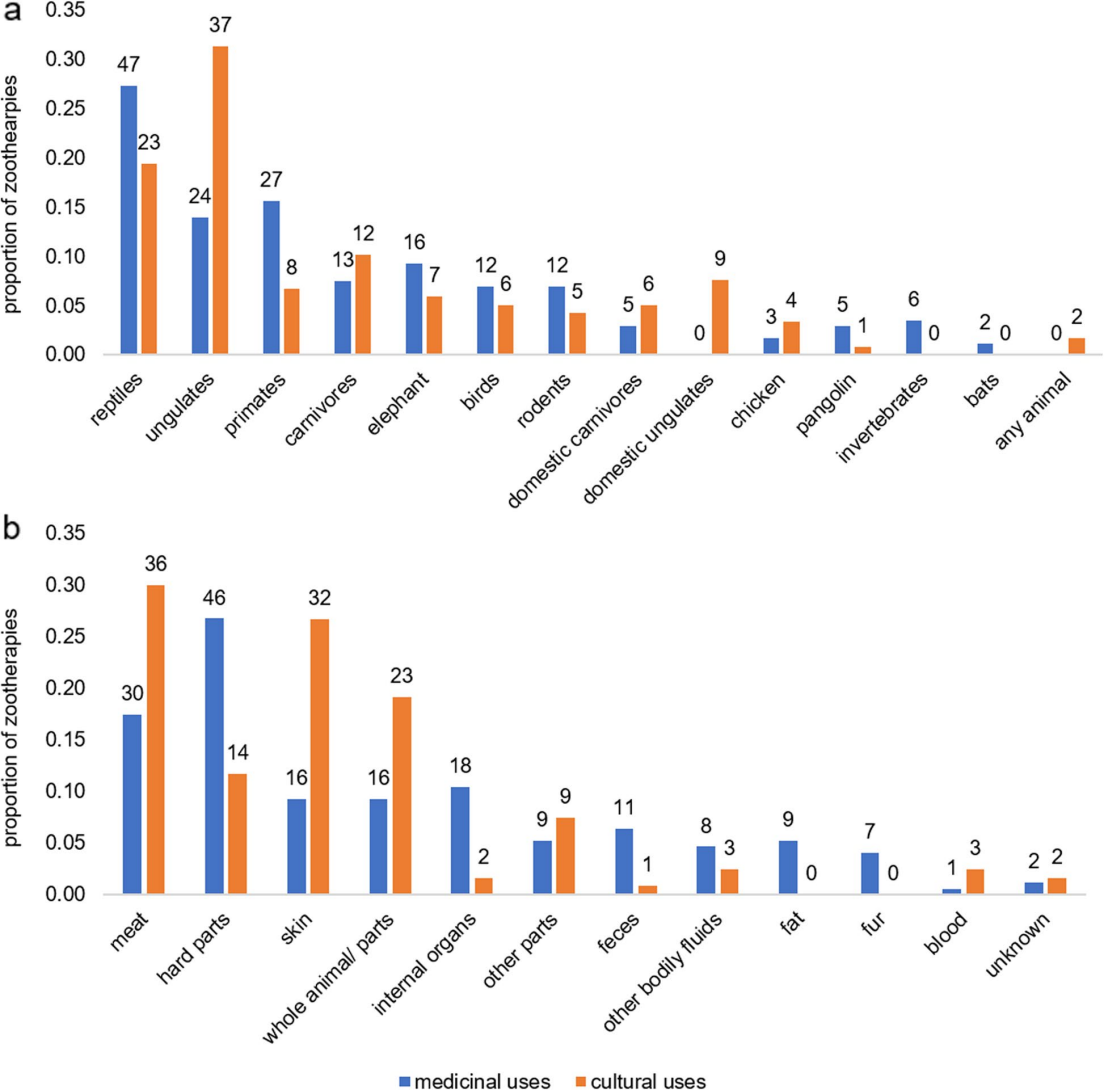
Proportion of described zootherapies, including medicinal (blue; *N* = 172) and cultural (orange; *N* = 120) practices, involving different taxonomic groups (**a**) and body parts (**b**)

Zootherapeutic practices involved a minimum of 44 different species (both domestic and wild), including endangered and critically endangered species (Fig. 1A; see Additional File 3 for taxonomic information). Animals known to be consumed locally, but that were not reported as useful for zootherapy included: golden cat (*Caracal aurata*), tree and rock hyraxes (*Dendrohyrax dorsalis* and *Procavia capensis*), giant otter shrew (*Potamogale velox*), African clawless otter (*Aonyx capensis*), mongooses (*Bdeogale nigripes* and *Crossarchus obscurus*), and unknown species of locally sourced and imported fish. Chameleons were the only animals used for medicine but not otherwise known to be consumed.

**Fig. 2:**
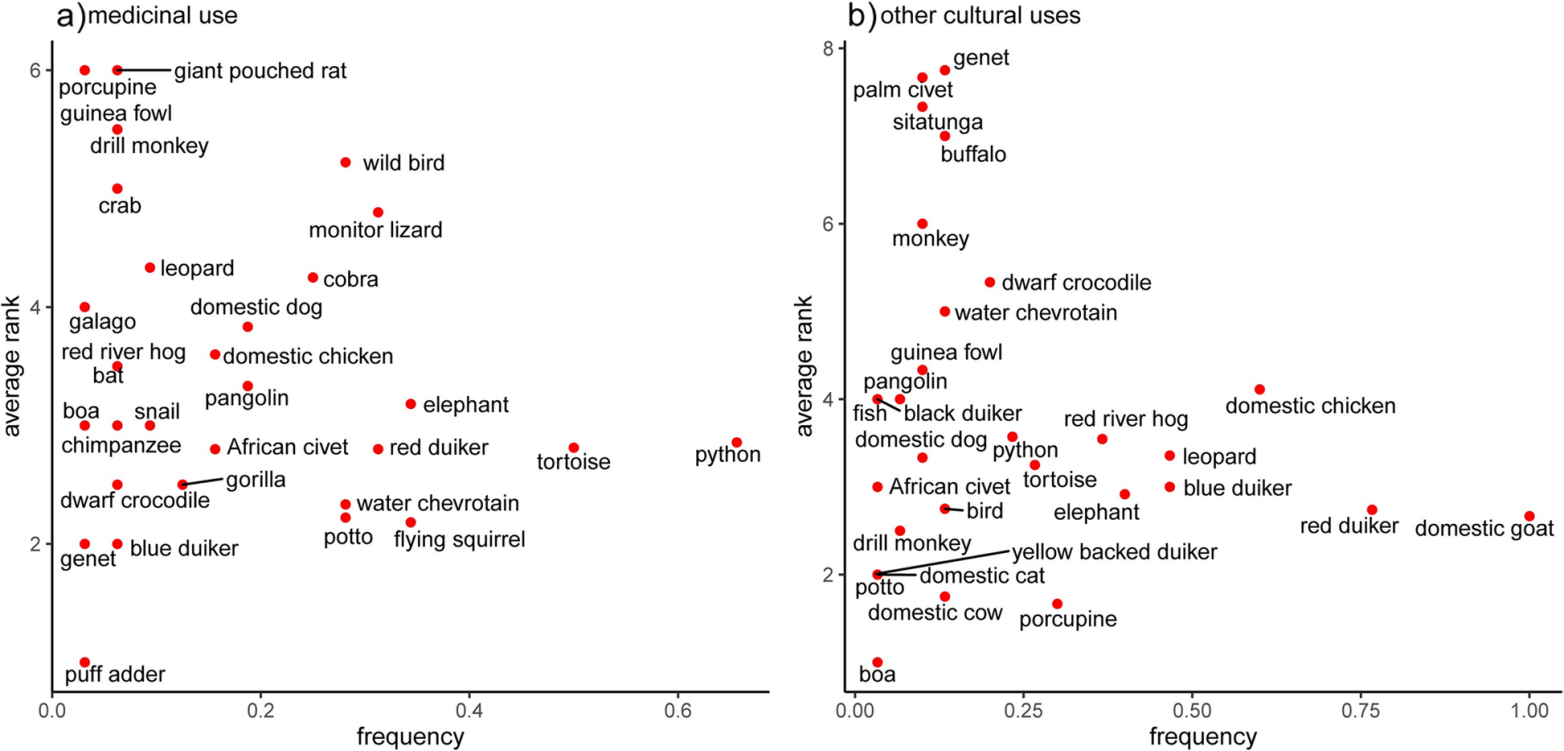
Salience of animals used for medicine (**a**) and other cultural purposes (**b**). Plots show the frequency at which an animal was mentioned (x-axis) and the average rank assigned to each animal (y-axis) during free listing exercises with key informants (*n* = 50). The most salient animals are shown in the lower right-hand quadrant, indicating they were frequently listed and assigned a high average rank (1 = most important)

The most medicinally salient animals (Smith’s S ≥ 0.05) included: python (*Python sebae*) (S = 0.17), tortoise (*Kinixya erosa*) (S = 0.11), flying squirrel (*Anomalurus beecrofti*) (S = 0.08), water chevrotain (*Hyemoschus aquaticus*) (S = 0.08), elephant (*Loxodonta cyclotis*) (S = 0.06), and potto (*Perodicticus potto or Arctocebus calabarensis*) (S = 0.05) (Fig. 2A; see Additional File 3 for specific practices). Goat (*Capra aegagrus*) (S = 0.20), red duiker (*Cephalophus dorsalis* or *C. ogilbyi*) (S = 0.17), porcupine (*Atherurus africanus*) (S = 0.07), chicken (*Gallus gallus*) (S = 0.07), blue duiker (*Philantomba monticola*) (S = 0.06), elephant (S = 0.06), and leopard (*Panthera pardus*) (S = 0.05) were considered highly salient animals for non-medicinal cultural practices (Fig. 2B; see Additional File 3 for specific practices).

### Animal parts, preparation, and administration

**Fig. 3:**
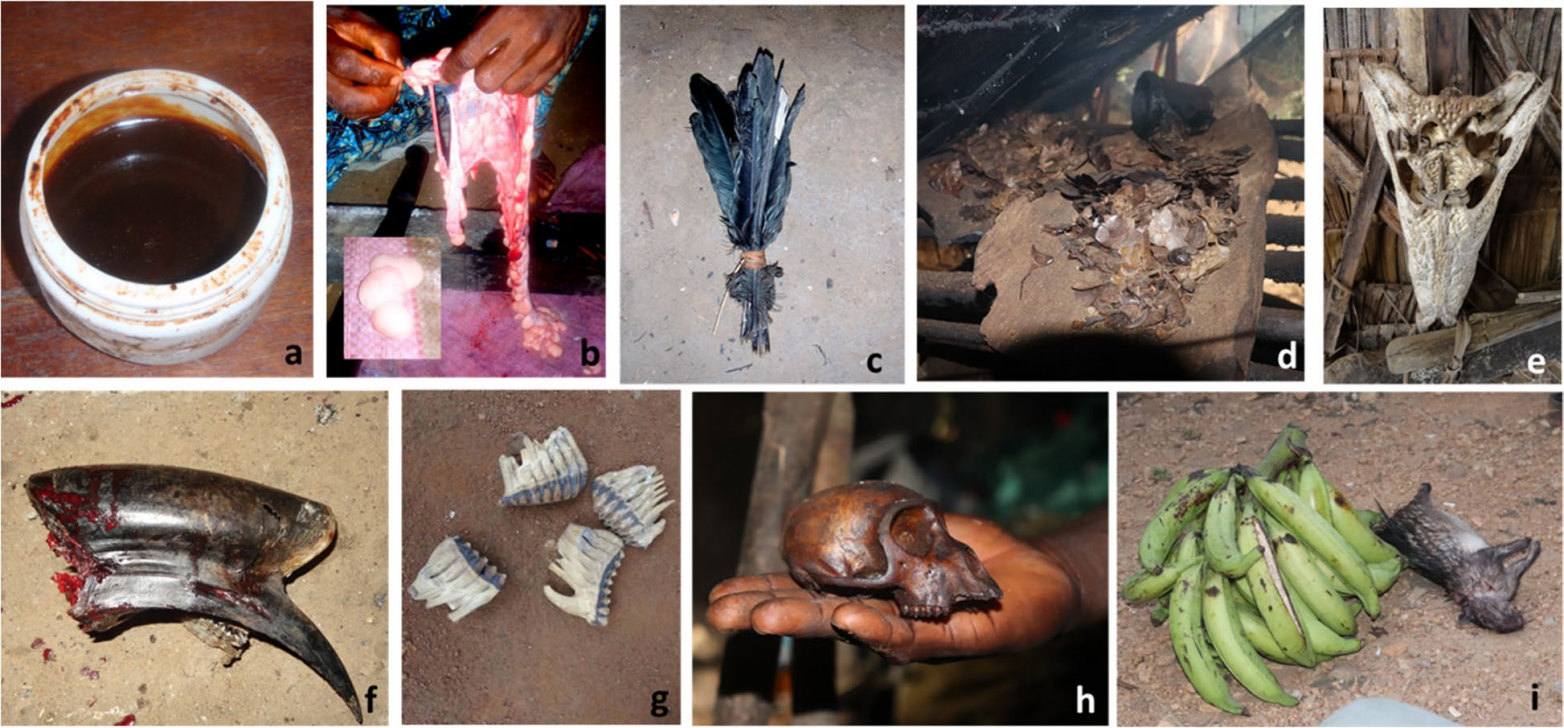
Images of animal parts used for zootherapy: python oil made from fat and stored for use as body rub (**a**); fresh python fat mixed with local liquor or dried and used as lozenges (**b**); wild bird feathers for various cultural practices and decorations (**c**); pangolin scales being reduced to ashes for consumption to cure various illnesses (**d**); skull of dwarf crocodile hung in town hall for use during cultural festivities and/or charms (**e**); hornbill beak (**f**) and elephant teeth (**g**) traded for unknown medicinal use; monkey skull used as a vessel to prepare and administer medicine (**h**); and whole raw porcupine gifted to visitors with plantain to welcome them and bring good luck (**i**)

Zootherapeutic practices involved the use of meat, hard parts (e.g., bone, skull, shell, beak, horn, scales, spines, teeth and tusks), skin, whole animals and whole animal parts (e.g., limbs or head, including skin, bone, and organs), internal organs (e.g., brain, gall bladder, intestine, kidney, pancreas, heart, scent gland, stomach, and gizzard), “other parts” (e.g. bone marrow, ears, eyes, tail, feather, whiskers, egg, and anus), feces, other bodily fluids (e.g., bile, stomach fluid, synovial fluid, and snake venom), fat, fur, blood, and unknown parts (Figs. 1B and 3; Table 1).

**Table 1:** Body parts used for zootherapy, taxonomic classification of animals used, and associated preparation and administration routes

Body part	#^a^	Taxonomic groups used	Preparation	Administration Route
**meat**	**68**	any animal, ungulate, domestic chicken, primate, reptile, domestic dog, carnivore, rodent, crab, domestic ungulate	direct heat	oral
		reptile, primate, ungulate	direct heat	oral (extract)
		primate, ungulate	direct heat	subcutaneous
		ungulate	direct heat	topical (skin)
		snail	raw	topical (eye)
		any animal, ungulate, carnivore, domestic ungulate, rodent, reptile, elephant, bird	raw or direct heat	non-specific contact
		ungulate	unknown	unknown
**skin**	**48**	carnivore, ungulate, domestic cat, reptile, primate, pangolin, elephant	passive heat	topical (skin)
		ungulate, rodent, carnivore, pangolin	passive heat	unknown
		reptile, domestic cat, carnivore, domestic ungulate	passive heat	non-specific contact
		reptile	passive heat	oral (extract)
		ungulate, carnivore, reptile	direct heat	oral
		reptile	direct heat	topical (skin)
		ungulate	direct heat	oral (extract)
		ungulate	direct heat	topical (inhaled)
**hard parts**	**42**	ungulate, primate, reptile, elephant, crab, rodent	dried (various mechanisms)	topical (skin)
		bird, reptile, elephant, primate, ungulate	dried (various mechanisms)	unknown
		primate, ungulate, reptile	dried (various mechanisms)	enema
		primate, ungulate, rodent, reptile	dried (various mechanisms)	subcutaneous
		ungulate, primate, snail, elephant	dried (various mechanisms)	non-specific contact
		domestic chicken, reptile, elephant	dried (various mechanisms)	no direct contact
		primate, reptile	dried (various mechanisms)	oral
		snail	dried (various mechanisms)	topical (eye)
		ungulate, pangolin, reptile	direct heat	oral
		reptile	direct heat	enema
		rodent	direct heat	topical (inhaled)
**whole animal/parts**	**41**	reptile, bird, primate, carnivore	unknown	unknown
		domestic ungulate, elephant, primate, rodent, ungulate, domestic chicken, reptile	raw or direct heat	non-specific contact
		domestic dog, domestic chicken	live	non-specific contact
		ungulate, domestic chicken	direct heat	oral
		reptile, pangolin	unknown	topical (skin)
		elephant, domestic dog	rinsed	enema (extract)
**internal organs**	**20**	bat, reptile, rodent, ungulate, domestic chicken	direct heat	oral
		reptile, primate, rodent	direct heat	oral (extract)
		reptile	direct heat	topical (skin)
		carnivore, reptile	raw	topical (skin)
		domestic cow	raw	non-specific contact
		rodent, reptile	liquor	oral (extract)
		bird, ungulate, carnivore	unknown	unknown
**other parts**	**20**	bird bone marrow, ungulate ear	direct heat	oral
		bird eye	unknown	unknown
		bird feathers	raw	topical (skin)
		bird feathers	raw	unknown
		carnivore whiskers	raw	oral
		domestic chicken egg	raw	non-specific contact
**feces**	**13**	ungulate, reptile, elephant, carnivore	dried or fresh	topical (skin)
		elephant, reptile	dried or fresh	subcutaneous
		carnivore	dried or fresh	enema
		elephant, primate	liquor	oral (extract)
**fat**	**11**	reptile	raw	topical (skin)
		domestic dog	direct heat	subcutaneous
		reptile, elephant	direct heat	oral
		reptile	unknown	unknown
**other bodily fluids**	**10**	reptile, elephant	liquor	oral
		elephant	raw	oral
		reptile	antivenom	intravenous
**fur**	**8**	bat, carnivore, primate, rodent	raw	topical (skin)
		rodent, primate	raw	oral
		domestic dog	raw	subcutaneous
**blood**	**4**	domestic dog, domestic chicken, domestic ungulate	raw	non-specific contact
		primate	raw	topical (skin)
**unknown**	**4**			

^a^Indicates the number of zootherapies a body part was used in

Zootherapies were prepared using live animals, raw animal parts and by-products, or by applying direct heat (e.g., cooking, boiling, drying, or melting under fire), passive heat (e.g., air or sun drying), or soaking in liquor (e.g., distilled palm wine) to animal parts and by-products (Table 1).

Consumption of cooked or dried meat from domestic and wild animals was commonly described for medicinal and cultural practices, with specific species being important under different circumstances (e.g., porcupine to welcome guests and potto to give strength to an unborn child (Additional File 3)). We found no examples of the use of raw meat for traditional medicine, except for the use of raw snail meat as an eye rub. Dried meat and organs were used to prepare powders, and raw internal organs were mixed with liquor to produce medicinal extracts. Some participants reported that water could be used when liquor was not available.

The preparation of meat, or whole animal/ whole animal parts, that were shared within the community for ceremonial purposes or good fortune was non-specific, as they could be offered as raw/dressed meat, smoked/dried meat, or as a whole animal (dead or alive). Zootherapies involving live animals were only described for domestic species, presumably due to the difficulty of procuring live wild animals from the forest. During live sacrifices, the person performing the sacrifice would slaughter the animal, occasionally making use of the raw blood. In contrast, when wild animals were sacrificed, they were conventionally hunted and butchered, and the sacrifice involved sharing pieces of dry meat with gods or ancestors by sprinkling them on the ground. Participants also described “washing” a live animal or whole animal parts and then using the leftover water as an enema for children.

Hard parts and skins were dried, either actively or passively, prior to their use in zootherapies. These parts were described as dried, but the same mechanism of drying was not consistently applied for a given use. For example, bones could be used after boiling or sun drying. Similarly, skins were prepared by either drying them in the sun (e.g., for use as cultural displays), or directly heating them via roasting over a smoking fire. Feces were used either fresh or dried, with dried feces being mixed with water prior to use as a rub, syrup, or enema. Animal fat was dried to prepare lozenges, or molten to prepare rubs or syrups. Blood and other bodily fluids were used raw to prepare rubs and tonics or mixed with liquor prior to consuming.

Animal parts were administered orally (direct or as an extract), topically (on skin, in eye, or inhaled), subcutaneously (in open cut or wound), as an enema, or were non-specific (Table 1). Orally administered zootherapies involved ingestion of food, broth, or drink made from animal parts, sucking on parts as a lozenge, or ingesting extract or mixture after an animal part had been mixed with liquor, water, or palm oil. Extracts or mixtures made from animal parts and by-products were also used as enemas for children. Topical applications included, wearing hard parts and skins, or using powders, molten fat, or feces as a body rub. Some treatments involved applying animal parts subcutaneously, by making incisions to an area before application or applying animal parts or by-products directly to an open wound. Contact with animal parts that were gifted, used as a vessel or instrument, or displayed in the house, were categorized as “non-specific contact”, as the ways in which people handled these items were not specified or consistent. For example, a person gifting meat as part of a cultural practice could be involved in hunting, butchering, and smoking meat prior to gifting, or could have meat sent to someone else without any direct contact.

Few zootherapies adhered to a common prescription. For example, we found numerous examples illustrating that for a single zootherapy, there may be variations that substitute different species (e.g., bat and galago fur instead of flying squirrel fur to treat burns and African civet, palm civet, and domestic cat skin instead of leopard skin), body parts (e.g., either blue duiker meat or organs used to cure stomachache), or preparation methods (e.g., elephant feces mixed with snuff, liquor, herbs, or used raw prior to application). Administration practices were also highly variable, for example, four different administration routes for gorilla bone were described by four different respondents who described it as useful for giving strength to a child, including: tying a bone to a child’s neck, grinding the bone for consumption, mixing ground bone with herbs to perform a ritual, or using ground bone in an enema. Other variations were also cited that involved the use of other primate species and parts to confer strength (e.g., drill monkey, chimpanzee, and potto). Details can be found in Additional File 3.

Animals and animal by-products used for zootherapy were procured by anyone in the community with the skills and tools to acquire them. We observed instances of people keeping animal parts and by-products, but not live animals, for later use in zootherapies. Medicinal treatments were then prepared by anyone with the knowledge of how to prepare and administer them. In contrast, many cultural practices, such as charms, were prepared only by juju doctors with specialized knowledge and power. Still, juju doctors often requested that customers bring the necessary animal parts to create charms, requiring users to procure the animal directly or sponsor a hunter to kill the animal on their behalf. We did not record any instances of wild animals being purchased from outside of the village (i.e., in case of extinction within the area).

### Limits to participant knowledge

Forty zootherapies included incomplete information regarding the parts used or how they were prepared or administered (Additional File 3). For example, participants reported using animals to provide protection against machetes and bullets, as aircraft charms that allow a person to be transported from one area to another, and for transformation into an animal, but they were either unable or unwilling to provide details on how the animals were used as these were held as trade secrets by juju doctors. Similarly, the details of animals used by secret societies, including sacrificial practices, were also kept secret by initiates. Trading in wild animal parts was another reason for incomplete knowledge, because “strangers” from other parts of Nigeria came to buy animal parts for medicine, but respondents themselves did not know the details of their use.

### Quantitative and qualitative differences in zootherapy users

Forty-five percent (*n* = 147) of 327 participants reported knowledge of use of wild animals for medicine, and 75% (*n* = 245) reported other cultural uses of wildlife. Nineteen percent (*n* = 62) reported using wild animals as medicine for themselves or their households. Use of wildlife for traditional medicine was positively associated with hunting for a livelihood compared to not hunting (OR = 2.47 [95% CI: 1.35–4.7]; *p* < 0.01). Age, family size, wealth, awareness of zoonotic disease, or village location in relation to the national park were not significantly associated with use of wildlife for medicine (see Additional File 4 for quantitative data). Men and women both provided information about treatments for both genders and different age groups. Qualitative analyses of zootherapies revealed seasonal as well as gender and age specific uses. For example, animals were used for medicine to treat women’s breast ailments, for maternal and antenatal care, to treat children’s cough, heat stroke, weakness, and to promote children’s intelligence. Animals were also used during cultural practices of male-only secret societies (e.g., practices of the leopard society), and for celebration of seasonal festivals (e.g., New Yam Festival) and treatment of seasonal ailments (e.g., catarrh or malaria). In at least one village, the celebration of the yam harvest involved ordering all men in the village to enter the forest and hunt in preparation for the festivities, regardless as to whether they were “hunters”.

## Discussion

Our study of hunting communities in Nigeria investigates zootherapy from a zoonotic risk perspective, examining not only which species people use for zootherapy, but how they use them (i.e., body parts used and preparation and administration practices). In addition, we analyze the sociocultural and environmental contexts of these interactions to achiever a deeper understanding of the role of zootherapy in a society in which it is practiced. Our findings reveal potential zoonotic risks, generate hypotheses for epidemiological and ecological investigations of zoonoses, and identify possible entry points for effective public health interventions to reduce human exposures to zoonoses through zootherapeutic practices.

### Species encounters via zootherapy

Zootherapies included use of both wild and domestic animals, including birds, rodents, primates, bats, ungulates, carnivores, reptiles, invertebrates, elephants, and pangolins. Animal groups and species varied in their importance in zootherapies, with wild animals most important for use in traditional medicine and domestic animals among the most important species used for other cultural purposes (Smiths S ≥ 0.05; Figs. 1a and 2b). These data show that practices related to zootherapy promote human contact with high consequence groups (rodents, bats, and primates), and that within these groups, nocturnal primates (potto/angwantibo) and porcupine were among the most culturally salient species used for medicine and other cultural practices, respectively.

Rodents were frequently reported as useful for zootherapy, offering potential routes for human exposures to rodent-borne zoonoses. Zootherapies included porcupine (family Hystricidae), giant pouched rats (genus *Cricetomys*), and flying squirrels (family Anomalure). Porcupines make up the majority of available mammal species sold as traditional medicine in markets across Africa [101] and are the preferred and most frequently hunted animals in our study region [15, 96]. Zootherapies included use of extract from raw porcupine heart soaked in liquor, consumption of porcupine intestine (which is not otherwise widely consumed due to its bitter taste [86]), and use of quills as a laceration tool (Additional File 3). Such uses of porcupine illustrate how animal parts that are raw or might otherwise be discarded, and administration practices that permeate the skin barrier, could lead to transmission of zoonotic agents. The existence of similar practices in other regions of Africa suggests any associated risks may be widespread; for example, porcupine intestine is also considered medicinal in Sierra Leone, when cooked in pepper soup (per obs., J. Bonwitt), and porcupine quills are used as a tool to puncture abscesses and boils in Tanzania [102]. Similarly, the zootherapeutic use of giant pouched rats (meat, intestine, and gall bladder) was reported in our study and others across Nigeria, suggesting widespread potential for exposure to zoonoses hosted by *Cricetomys* spp. [64, 103–106].

Porcupine and giant pouched rats are both possible reservoirs for monkeypox virus, which is currently re-emerging in Nigeria with poorly understood zoonotic origins [107–110]. However, we are unaware of the occurrence of monkeypox or other zoonoses hosted by porcupines or giant pouched rats within our study villages. *Cricetomys* species and Old World porcupines have also been found to be infected with nairoviruses, the genus of bunyaviruses that includes Crimean Congo Hemorrhagic Fever [34, 111], and porcupines have been associated with anthrax outbreaks in other parts of West Africa [112]. Flying squirrels (family Anomalure) were among the most important species used for medicine. Flying squirrel fur was especially useful for treating burns (Additional File 3), again, paralleling zootherapies described in Sierra Leone [16] and suggesting extensive use of this zootherapy across West Africa. While other small rodents (e.g. mice and rats) are important household and agricultural pests throughout West Africa, they were not frequently consumed within our study communities [96], nor were they listed as useful for medicine or other cultural purposes. However, practices that might present a risk of zoonotic exposures to small rodents elsewhere include consumption of the intestine of the brush-tailed rat to ease stomach pain [16], storing newborn mice in oil that is topically applied to wounds [43], and chewing a concoction of rodent feces wrapped in special leaves or eating of food leftover by rats to ease childbirth [113, 114]. Epidemiological investigations of rodent-borne zoonoses should therefore explicitly consider zootherapy among possible routes of zoonotic exposures.

Primates were the second most cited group for medicinal uses, and the third most common for zootherapies generally (Fig. 1A). Zootherapies involving non-human primates offered points of contact with body parts that were not typically consumed, including bones, feces, hands, skin, blood, and fur. The use of chimpanzee blood, bone, and meat were reported to confer strength by participants of this study (Additional File 3) and in Sierra Leone (per. obs., J. Bonwitt). Several studies have identified multiple simian retroviruses that are transmissible to humans in West and Central Africa from blood exposure [67, 115, 116], however, examination of risk factors for primate exposure and retroviral transmission have predominantly focused on hunting, butchering, and consumption of primates [117–119]. Our data demonstrate plausible routes for exposure to simian viruses that extend beyond the hunting and consumption of bushmeat.

Bats were used as traditional medicine in our study population, including topical application of fur to treat burns and consumption of cooked brain to enhance children’s intelligence (Additional File 3). Although bats were generally ranked as low importance for their zootherapeutic value, the use of bat heads for zootherapy may introduce novel risks, as a diversity of lyssaviruses with neurological tissue tropism have been identified in bats of sub-Saharan Africa [120]. Similar reports from traditional healers in Senegal revealed frequent use of both heads and whole bodies of the Rüppel’s Horseshoe bat (*Rhinolophus fumigatus*) in potions brewed for the treatment of mental illness [121]. Other zootherapeutic uses of bats described elsewhere include the inhalation of smoke from burning bats to treat pneumonia in Tanzania [102] and consumption of bat meat to improve female fertility and celebrate religious festivals in other regions of Nigeria [64, 65]. Bats are also sold in traditional medicine markets by 49% of traders in Benin [44] and 21% of traders in South Africa for “unknown reasons” [101].

Contacts with domestic animals via zootherapy were qualitatively different from contacts with wild animals in that contact with domestic animals were more likely for cultural (vs. medicinal) practices, involved contact with live animals (e.g., live sacrifices), and were more likely to involve animal blood. Domestic animals were also used as replacements for wild animals when these were not available, suggesting that the use of domestic animals in zootherapy may rise as environments becoming increasingly human dominated. Although zoonoses from domestic animals are less likely to emerge than zoonoses from wildlife [122], domestic species host many zoonotic pathogens and can play important roles as intermediate hosts of wildlife-origin pathogens [35, 123]. Future studies should therefore explore the use of zootherapy to cure animal diseases, given that traditional healers are also important parts of veterinary health care systems across developing countries [124], and domesticated animals can act as bridge or amplifier hosts to facilitate transmission of pathogens of wildlife-origin to humans.

Other taxonomic groups used for zootherapy included pangolins, elephants, and reptiles, as well as domestic and wild birds, carnivores, and ungulates. Pangolins were used locally and were traded for unknown zootherapeutic purposes. Pangolins are also used widely for zootherapy in other regions of Nigeria [125, 126], other African countries [127, 128], and globally [129]. Recent characterization of coronaviruses similar to SARS-CoV-2 in pangolins in Asia suggests potential emerging infectious disease risks associated with pangolin trade [130]. Furthermore, trade in pangolins for their medicinal properties has contributed to the near extinction of Asian pangolins, and redirected attention to African pangolins to meet demand [131], highlighting the potential zoonotic significance of human-pangolin interactions through zootherapy at multiple geographic scales.

In Nigeria, zootherapy often extends to animals not typically used for human consumption, including amphibians, skinks, shrews, small birds and rodents, and some insects [7]. However, we only identified one animal, the chameleon, used for medicine, but not otherwise consumed. While no species was considered universally taboo in our study communities, some family rules forbid consumption of certain species due to family mythologies [15]. Nevertheless, studies have shown that people will often kill, sell and/or use medicinal concoctions of animals that are undesirable or taboo to eat [7, 16, 38]. In such cases, taboos do not necessarily preclude their use among the wider community, and interactions with such species through zootherapy can remain a source of zoonotic infection. Focusing on hunting for consumption alone can therefore blind researchers to human-animal interactions and potential zoonotic exposures through zootherapy.

### Zootherapeutic practices that can modify exposure risks

Our data provide insight into zootherapeutic prescriptions, including preparation and administration practices, that may increase or decrease the risk of potential exposure to zoonotic pathogens. Use of animal parts and by-products for zootherapies that would otherwise be discarded creates unique forms of interspecies interactions that may generate zoonotic exposures. For example, the use of feces, especially from primates, in many medicinal concoctions could facilitate the spread of enteric pathogens with infective stages that are shed in feces [132, 133], whereas the use of chimpanzee blood could facilitate transmission of blood-borne pathogens, such as simian retroviruses. The practice of using animal brains and skulls may generate exposure to lyssaviruses, which are able to infect brain tissue [120]. In addition, therapies that make use of dried animal parts (e.g., powders or animal skins) may pose a unique risk for zoonotic pathogen exposures. Animal skins have been implicated as a source of anthrax spores in endemic countries and through trade in animal parts [134], and other pathogens such as orthopoxviruses, including monkeypox, can have lengthy environmental survival in tissue [135]. Our data highlight that low-utility animal parts that do not feature prominently in diets are still handled within communities via zootherapeutic practices, creating under-investigated exposure risks.

Exposure risks may be mitigated or exacerbated by preparation and administration practices, which are not widely reported in zootherapy research. For example, practices that heat inactivate or soak animal parts and by-products in alcohol prior to their administration may reduce exposure risks. Time and temperature needed for pathogen inactivation is significantly affected by the type of microorganism and its location (e.g., tissue vs. excreta) [136]. Given the variation in heating techniques (e.g., boiling, smoking, sun-drying) applied to a wide diversity of animals and animal parts, we expect higher variability in exposure risks associated with handling animals for zootherapy compared to hunting and consumption. Similarly, the use of local alcohol (traditionally, distilled palm wine) to prepare zootherapies could help to inactivate some pathogens. However, disinfecting properties of locally produced alcohol are unknown. A 60–70% alcohol solution is recommended for sterilizing contaminated objects in healthcare settings [137]. Studies from Nigeria show that locally distilled palm wine ranges from 41 to 78% alcohol [138], though the range is likely larger due to variation in distillation methods as well as the common practice of diluting local liquor with water. Even at high alcohol levels, liquor may be ineffective in deeper tissues, and reports of substituting liquor with water would negate any possible risk reduction associated with this practice. Importantly, preparation norms that may reduce risks to end-users’ would not decrease exposure risks in individuals procuring and preparing animal products.

Zootherapeutic administration practices included subcutaneous, topical, inhalation, oral, and anal exposure routes that alter the way that animal products and potentially infectious pathogens can enter the body. Parenteral routes (i.e., injection) may increase infection risks beyond what would be expected from hunting and consumption of animal products. For example, the common practice of making incisions is likely to enhance infection risks from zoonotic pathogens as well as general infection from use of non-sterile products. Inhalation routes were described for zootherapies involving ungulates and rodents, which are known to transmit anthrax and hanta- and arena viruses, respectively. Rodent-borne hantaviruses and arenaviruses can be transmitted when virus particles are aerosolized on dust particles, such that people are advised to avoid raising dust to reduce chances of breathing it in [139, 140]. Variation in administration practices could in turn affect the type and severity of disease; for example, the illness associated with anthrax varies depending on how it enters the body, with inhalation anthrax considered to be the deadliest form [141].

In our study, relatively few zootherapies included administration of live animals, fresh parts, raw meat, or blood, suggesting that risks to end-users are low relative to those who procure and prepare zootherapies. While people will commonly keep live animals for pets, food, or trade, we did not observe any instances of live animals being kept alive for local zootherapeutic uses. The use of animal blood was reported in only a few descriptions of zootherapies (domestic animals and primates). Still, hunting and butchering of animals during or prior to their preparation for zootherapy involves extensive contact and environmental contamination with animal blood. It was difficult to pinpoint where blood exposures related to zootherapy might occur, because animals could be obtained by either hunters, healers, or end-users, and prepared by friends, family members, designated healers, or users. Future studies examining zootherapy value chains would be better suited to determine product preparation to further assess the risk of zoonotic exposures across these groups.

### Sociocultural and environmental contexts of zootherapeutic practice

Nineteen percent of participants reported using zootherapy for themselves and their family, which is similar to other regions within Sub Saharan Africa (23% in Uganda [142]). Use of zootherapies was patterned by livelihoods, with male hunters significantly more likely than male non-hunters to use animals for medicine for themselves and their family, perhaps due to ease of access to and enhanced knowledge of animals. Village proximity to the forest was not associated with the likelihood that individuals used zootherapies, despite observed differences in wildlife consumption between these areas [86]. We found no association between use of zootherapies and zoonotic disease awareness. However, perceptions of zoonotic disease risk may have affected use of zootherapies in ways that were not measured (e.g., use of species or handling norms). Furthermore, zoonotic disease awareness (53% of participants in 2012 [15]) likely increased following the 2014 Ebola Virus Disease outbreak. Nevertheless, members of the study community anecdotally reported that such diseases are not found in their community, as they have been hunting for many generations without getting sick. Together, these data show that zootherapy is widespread in our study region, but that increased use of animal products by hunters and their families may further contribute to heightened zoonotic exposure risks associated with hunting as an occupation. Zoonotic disease awareness programs in areas that have not experienced outbreaks may be minimally effective in risk reduction.

Several zootherapeutic uses of animals were specific to certain groups (i.e., men, women, and children) or seasons, which could further pattern exposure risks. For example, some administration practices (e.g., enemas) and contact with certain animals and body parts (e.g., primate feces and bat brain) were limited to zootherapies that treated children. Other animals, including non-human primates and red duiker, featured prominently in zootherapies for prenatal care. Seasonal patterns (e.g., use of animals to treat seasonal infections such as malaria, cold/catarrh, and heat stroke, or for celebration of annual festivals or holidays) may further heighten contact in certain times of the year. Such information could be used for deciphering the epidemiology of zoonotic diseases and/or targeting interventions, for example, to hunters or including messaging about zootherapies in maternal healthcare programs.

Salient practices were difficult to define within our study area due to high variability in parts used and their preparation and administration. Indeed, traditional medicinal practices can be highly variable when: people practice do-it-yourself home remedies, treatments have high failure rates, practitioners compete with one another using different curative techniques, or treatments are prescribed within transitioning healthcare systems [143]. The value of traditional remedies can lay in their originality, which serves to conceal uncertainty in their effectiveness and lack of knowledge of those who prescribe and prepare zootherapies. High failure rates characteristic of traditional medicine can thus paradoxically encourage, rather than prevent, proliferation and diversification of cure. Variability in zootherapeutic practices may also reflect transitioning health systems that borrow from both local and global knowledge, combining “traditional” and “conventional” biomedical practices to meet the needs of local health systems and wildlife trade networks. In all, these results contribute to a growing number of examples showing that the practice of zootherapy in Nigeria is highly variable and lacks formulated standards or universal protocols [6, 7, 105, 144].

Our focus on people who prescribe, prepare, administer, and use zootherapies within the communities that source these animals provided a level of detail that is not always achievable in other settings, for example, trade markets. Like wildlife hunting and consumption, explorations of zootherapeutic uses of wildlife have largely focused on market surveys [103, 105, 144–147], which provide tallies of the species that are used, but offer limited information on the nature of their use due to limits in knowledge of traders who act as middlemen between hunters and end-users. Additionally, market surveys can miss species that are used locally but not widely traded [148]. We were unable to determine details of animal use in zootherapies when people did not know, did not care to know, or did not want to reveal details of their practices. Participants reported not knowing the use of specific animals and animal parts that were traded to people from other regions of Nigeria. This suggests that, unlike studies where indigenous knowledge is borrowed for use and trade [149], traders source animals for medicine, but not knowledge of local practices, from participants of our study communities. People may also keep certain details secret when protected species are used, patients do not want to reveal stigmatizing conditions (whether medicinal or related to witchcraft) [150, 151], or traders do not want to expose trade secrets [101, 152, 153]. Though we tried to circumvent some of these challenges by asking about community-wide uses of animals, rather than exposing individual behavior, it is important to recognize that secrecy is embedded within traditional medicine, and there are inherent limits to the knowledge that we were able to obtain. Additionally, by focusing on community wide patterns, we captured a wide diversity of practices, but were unable to determine the frequency of use of these animals by individuals. This presents a challenge to identifying salient forms of zoonotic risks and developing interventions that target these practices.

Our data revealed some principles that may guide prescriptions of zootherapies in our study region and pattern an otherwise seemingly diverse set of practices. For example, some uses were based on bioactive components of animals (e.g., bile) or physical attributes of body parts (e.g., use of bones to give strength). Evidence from other studies shows that the condition of an animal (e.g. alive or dead) is sometimes determined by the raw materials to be extracted and the type of illness that is being treated [98]. Our results show common practices associated with different animal parts; for example, wearing or preparing powders from hard parts, using fat and feces to prepare body rubs, and ceremonial consumption of cooked meat. The therapeutic application of animal fat is particularly widespread [11, 43, 149, 154–156], and an ethnopharmacological study (of anaconda fat in the Amazon) suggests some fats may have significant anti-inflammatory effects [154]. Interestingly, the authors identified oil extracts from native plants with similar fatty acid compositions that could offer a potential alternative to animal fat. Public health messaging targeting administration practices for different animal parts, and providing potential alternatives to these parts, may therefore be more effective than those that focus on specific prescriptions.

We found evidence that zootherapies were informed, in part, by sympathetic healing (i.e., animal characteristics resembling or symbolically associated with the condition were sometimes described as useful for treating it). For example, zootherapies included the use of skin and bones from “strong” animals for giving strength, treating broken bones, and making bullet proof charms or use of birds in making aircraft charms (Additional File 3). Indeed, the use of bones for osteological medicine is commonly practiced across different societies [11, 157]. Pottos were used to promote strength of an unborn child when used by the pregnant mother [148]. These animals are known for their toughness and strength, with hunters reporting having to pry the animals off of tree branches after killing them [158]. Indeed, aptitude transfer, in which the species used confers strength or attributes of that species is believed to enhance health and social lives of people in other regions [149, 155]. The use of primate feces to cure cough was justified through observations of seeing monkeys cough in the forest; in this region primates can serve as hosts for a zoonotic lung fluke that presents clinically with a cough [159]. Sympathetic healing also shapes contact with high risk taxa in other areas; for example, pregant women consume remnants of food leftover by a rat to ease labor [113]. Similarly, heads and whole bodies of Rüppel’s Horseshoe bat were used in traditional medicines for the treatment of mental illness because bats exist as a symbol of orientation, and therefore could aid patients who by the healer’s diagnosis lacked mental orientation [121].

Our data also provide evidence of “like cures like” theory of healing, in which a substance capable of causing an illness or injury is also capable of curing it (e.g., the use of dog saliva to cure dog bites and snake teeth to cure snake bites). Similarly, python flesh placed into local liquor was used to prevent people from turning into a python and the bile of the python could be used to create and cure poison, depending on its preparation. Thus, while individual uses of animals were highly variable, there were patterns governing the underlying logic of their use. Indeed, the diverse use of zootherapies presents a major challenge to generalizable inclusion within public health messaging and interventions. Targeting the reasons for use of animals in medicinal and cultural practices, as opposed to specific zootherapies, may therefore be a more productive entry point for public health interventions.

Our results show that human-animal interactions with species via zootherapy are not static in space and time, as use of certain animals is subject to availability of ingredients within ecological and cultural settings, and in relation to broader trade networks, and socioeconomic constraints [160]. For example, early ethnographic accounts from our study region describe uses of animals such as leopards and manatees for zootherapy in this region of Nigeria [93], however; species declines now prohibit their availability for such uses. Indeed, we identified high plasticity in zootherapeutic practices, including several examples of zootherapies involving the replacement of rare animals with more common or domestic species (e.g., replacement of leopard parts with those from other wild and domestic cats). The use of bat fur to cure burns also appeared to be an alternative to the more common practice of using flying squirrel fur. Similarly, we found evidence of utilitarian redundancy, in which some species (including from “high consequence” taxonomic groups) were utilized for the treatment of more than one disease or symptom [151, 156]. These data suggest that species that do not have specific zootherapeutic use at a certain place and time may have been used in the past or may act as a substitute for similar animals in the future.

Such adaptive responses to changing environments suggest that animal products from more abundant species, including domestic animals, could replace species of conservation or health concern. However this trend does not appear to be universal [161]. More research is needed to determine if species replacements would be acceptable even when preferred options are still available. Zoonotic reservoir hosts are often among some of the most ecologically resilient species [162, 163], and as environments become increasingly human-dominated, replacements could lead to an increase in contact with disease reservoirs through zootherapy. Plasticity in use of animal ingredients therefore highlights interesting interactions between conservation and health that have yet to be thoroughly explored. This is especially important in areas under constant human activities, such as deforestation, agricultural expansion, and hunting, that construct risky human-animal interfaces (e.g., [162]).

### Study limitations

Our study used a mixed-methods approach that focused primarily on community-wide practices to capture the breadth of zootherapies used within a single region and details of their preparation and administration. In doing so, we were unable determine the frequency by which certain products were used and how widespread their use was within and between populations. Future studies focusing on prevalence and frequency of use of different species and body parts, variation in preparation and administration practices, and who prescribes zootherapies across different subsets of society (e.g., gender and generation-based differences) will be important for deciphering potential zoonotic risks within these populations. Although zoonotic diseases such as monkeypox, Lassa fever, Ebola Virus Disease, and anthrax are known to affect eastern Nigeria and other West African tropical rainforest communities, we do not have data on the presence of these zoonoses within our specific study communities, which have limited access to formal healthcare or diagnostic centers. During interactions with community members and local healthcare workers, we learned of skin rashes and fevers that do not respond to medication (e.g., anti-malarial drugs), and for which the origins remain unknown. Interviews with local health care workers and surveillance for zoonotic diseases within our study population would be helpful in further assessing risks involved with the various practices. Animal surveillance is needed to designate animals in these areas as reservoirs of zoonotic disease, and to provide direct evidence on the risks associated with different animal parts and practices. This will complement studies that aim to further understand the nature and contexts of human-animal interactions for assessing exposure risk. This paradigm has yielded a substantial body of research and knowledge surrounding hunting and consumption practices (e.g., [15, 16, 117, 164]), but insights into less visible forms of contact (i.e. zootherapy) have remained limited. Our mixed methods study provides a framework for examining practices in the context of zoonotic risks and generates hypotheses that will help guide surveillance for, and epidemiological investigations of, zoonotic pathogens.

## Conclusion

Our study demonstrates the importance of understanding zootherapeutic practices as risk factors for zoonotic diseases. In this way, traditional knowledge can help investigate One Health challenges. Our findings support previous calls to extend our understanding of the human-wildlife interface and zoonotic disease risk beyond bushmeat and bushmeat hunters [3, 165]. Epidemiological investigations and research into animal-borne pathogens should broaden efforts to consider medicinal and cultural practices that involve human-animal contact. Furthermore, public health messaging about infection prevention and zoonotic transmission should be explicit in their inclusion of medicinal and other cultural uses of wildlife. Although responding to the economic and nutritional costs incurred from hunting bans is already challenging [166], the cultural uses of animals in traditional medicine and spiritual practices may be even more difficult. Interventions should therefore recognize cultural importance of zootherapies and focus on culturally acceptable risk mitigation, rather than curtailing or halting the practices themselves. Overall, the study of zootherapies, and design of public health interventions that incorporate the use of zootherapies to prevent zoonotic transmission, will require mixed methods approaches and the efforts of social scientists alongside epidemiologists and health practitioners.

## Supplementary Information

**Additional file 1: S1**. Criteria and strategy used to look for articles investigating zootherapy from a zoonotic risk perspective.

**Additional file 2: Table S1**. Participant demographics, recruitment, and study design for research carried out during two study periods.

**Additional file 3: Table S2**. Medicinal uses of animals. **Table S3.** Cultural uses of animals.

**Additional file 4.** Quantitative Data. (CSV 7 kb)

## Data Availability

All data generated or analyzed during this study are included in this published article [and its supplementary information files].
