# Supplementary material for: Zootherapy as a potential pathway for zoonotic spillover: a mixed-methods study of the use of animal products in medicinal and cultural practices in Nigeria
Source: One Health Outlook. 2022 Feb 26;4:5. doi: 10.1186/s42522-022-00060-3 (PMC8881094; doi:10.1186/s42522-022-00060-3)
Supplement: Supplementary file 1 — Additional file 1: S1. Criteria and strategy used to look for articles investigating zootherapy from a zoonotic risk perspective. [file 42522_2022_60_MOESM1_ESM.docx]

**S1. Criteria and strategy used to look for articles investigating zootherapy from a zoonotic risk perspective**

**PubMed**

((zoono*[Title/Abstract]) OR (pathogen*[Title/Abstract]) OR ("disease reservoir"[Title/Abstract]) OR (parasite[Title/Abstract]) OR (virus[Title/Abstract]) OR (bacteria[Title/Abstract])) AND ((zootherap*[Title/Abstract]) OR (ethno*[Title/Abstract]) OR ("folk medicine"[Title/Abstract]) OR ("folk remedies"[Title/Abstract]) OR ("home remedies"[Title/Abstract]) OR ("indigenous medicine"[Title/Abstract]) OR ("primitive medicine"[Title/Abstract]) OR ("traditional medicine"[Title/Abstract]) OR ("medieval medicine"[Title/Abstract])) AND ((survey[Title/Abstract]) OR (ethnography[Title/Abstract]) OR (questionnaire[Title/Abstract]) OR (ethnology[Title/Abstract]) OR (review[Title/Abstract]) OR ("Knowledge, Attitudes, and Practice"[Title/Abstract]) OR (KAP[Title/Abstract])) AND (animal[Title/Abstract])

**Web of Science**

((zoono*) OR (pathogen*) OR ("disease reservoir") OR (parasite) OR (virus) OR (bacteria)) AND ((zootherap*) OR (ethno*) OR ("folk medicine") OR ("folk remedies") OR ("home remedies") OR ("indigenous medicine") OR ("primitive medicine") OR ("traditional medicine") OR ("medieval medicine")) AND ((survey) OR (ethnography) OR (questionnaire) OR (ethnology) OR (review) OR ("Knowledge, Attitudes, and Practice") OR (KAP)) AND (animal)

**JSTOR (200-character restriction)**

((zoono*) AND ((zootherap*) OR (ethno*) AND ((survey) OR (ethnography) OR (questionnaire) OR (ethnology) OR (review) OR ("Knowledge, Attitudes, and Practice") OR (KAP)) AND (animal)
