# Supplementary material for: Zootherapy as a potential pathway for zoonotic spillover: a mixed-methods study of the use of animal products in medicinal and cultural practices in Nigeria
Source: One Health Outlook. 2022 Feb 26;4:5. doi: 10.1186/s42522-022-00060-3 (PMC8881094; doi:10.1186/s42522-022-00060-3)
Supplement: Supplementary file 2 — Additional file 2: Table S1. Participant demographics, recruitment, and study design for research carried out during two study periods. [file 42522_2022_60_MOESM2_ESM.docx]

Table S1. Participant demographics, recruitment, and study design for research carried out during two study periods

|  | 2012 | 2017 |
| --- | --- | --- |
| Villages (n= no. of sites) | 5 | 6 |
| Ejagham villages | 1 | 3 |
| Ayo villages | 3 | 1 |
| Boki villages | 0 | 2 |
| Idoma villages | 1 | 0 |
| Sex (n= no. of participants) |  |  |
| Male | 327 | 34 |
| Female | 0 | 16 |
| Recruitment strategy | Purposive sampling of adult male hunters; randomly sampled adult male non-hunters | Purposive sampling of individuals with specialist knowledge |
| Study tools | Quantitative surveys and field notes | Structured key informant interviews and field notes |
