# Supplementary material for: Zootherapy as a potential pathway for zoonotic spillover: a mixed-methods study of the use of animal products in medicinal and cultural practices in Nigeria
Source: One Health Outlook. 2022 Feb 26;4:5. doi: 10.1186/s42522-022-00060-3 (PMC8881094; doi:10.1186/s42522-022-00060-3)
Supplement: Supplementary file 3 — Additional file 3: Table S2. Medicinal uses of animals. Table S3. Cultural uses of animals. [file 42522_2022_60_MOESM3_ESM.docx]

**Table S2.**  Medicinal uses of animals.

| **Year** | **#** ^1^ | **Body part** | **Treatment** | **Preparation and administration**^2^ |
| --- | --- | --- | --- | --- |
| **African civet (***Civettictis civetta*) | | | | |
| 2017 | 5 | anus | stomach pain | unknown (2) |
|  |  | fur | burns | applied to affected area (1) |
|  |  | scent gland | unknown | dried and sold to people (Hausa) who use it for medicine (1) |
| 2012 | 2 | scent gland | dislocation, body ache | used as rub (1) |
|  |  | flesh (leg) | child's heat stroke | ash from dried leg mixed with oil and drank by child (1) |
| **Bat** (*Chiroptera*) | | | | |
| 2017 | 2 | brain | enhances intelligence in children | brain is cooked and given to children above the age of six months (1) |
|  |  | fur | burn | applied to affected area (1) |
| **Blue duiker** (*Philantomba monticola*) | | | | |
| 2017 | 2 | head | headache | head is cooked together with some herbs and then consumed (1) |
|  |  | bone (skull) | headache | head is cooked, skin is removed, and skull is rubbed on the forehead (1) |
| 2012 | 32 | bone (skull) | headache | skull used as cup to drink from (2) |
|  |  | bone (skull) | headache | skull used to keep palm oil for rub (1) |
|  |  | leg (bone and flesh) | headache | ground, sometimes mixed with water, and rubbed on forehead (26) |
|  |  | flesh | stomach pain | mixed with herbs (peppe soup) and consumed (1) |
|  |  | intestine | stomach pain | mixed with herbs (peppe soup) and consumed (1) |
|  |  | skin | multiple | roasted and mixed with medicine and oil, used as lozenge (1) |
| **Boa** (*Calabaria reinhardtii*) | | | | |
| 2017 | 1 | fat | stiffness/ old injury | rubbed on affected area until it softens (1) |
| **Chameleon (***Chamaeleonidae*) | | | | |
| 2012 | 2 | skin | epilepsy, mental illness | roasted, ground, and rubbed on body (2) |
| **Chicken** (*Gallus gallus*) | | | | |
| 2017 | 5 | flesh | anemia | consumption of chicken pepper soup used as blood tonic (1) |
|  |  | whole animal (rooster) | many sicknesses | traditional healers use for many medicine (1) |
|  |  | bone | fracture and dislocation | an injury is given to the animal at the same body part where the patient is injured, then as the chicken is treated, the patient heals mysteriously (1) |
| **Chimpanzee** (*Pan troglodytes ellioti*) | | | | |
| 2017 | 2 | bone | weakness | child wears the bone as a sign of strength (2) |
|  |  | bone and blood | weakness | bone and blood mixed with chalk and rubbed on leg to improve strength (1) |
|  |  | flesh | weakness in unborn child | eaten during pregnancy to confer strength of animal to unborn child (1) |
| 2012 | 1 | bone (middle finger) | gives strength | dried and mixed with native chalk, used as rub for child (1) |
| **Cobra** (*Naja melanoleuca*) | | | | |
| 2017 | 8 | bile | poison | when added with another [unspecified] ingredient and consumed, the bile will cure poison (1) |
|  |  | flesh | joint pain | cooked and consumed (1) |
|  |  | skin | stomach pain | dried and soaked in water, then the extract is given to the patient orally (1) |
|  |  | venom | snake bite | venom used to produce antivenom (1) |
|  |  | head | unknown | sold to Yoruba people (1) |
| 2012 | 1 | flesh | unknown diseases | cooked and consumed (1) |
| **Crab** (*Potamonemus* sp.) | | | | |
| 2017 | 2 | flesh (abdomen) | bed wetting | cooked and consumed (1) |
|  |  | flesh (abdomen) | childbirth | cooked and consumed to ease childbirth (1) |
|  |  | shell | severe rashes | shell is dried and ground together with white chalk (limestone) and the powder is applied to the affected part (1) |
| **Dog** (*Canis lupis*) | | | | |
| 2017 | 6 | flesh | malaria, fever | meat is prepared in a soup and consumed (3) |
|  |  | fat | dog bite | fat is extracted when a dog is cooked and then stored (called boa oil), when needed it is mixed with alligator pepper and rubbed into bite wound (2) |
|  |  | flesh (leg) | weakness in unborn child | legs are cooked and consumed by pregnant women to give strength (1) |
|  |  | live animal | weakness or walking problems in a child | wash the limbs of a living animal with water, the resulting water is used to "pump" (give enema to) a child (1) |
|  |  | fur | dog bite | fur of the dog that bit you is placed on the wound (1) |
| **Drill Monkey (***Mandrillus leucophaeus*) | | | | |
| 2017 | 2 | bone (thigh) | waist pain or walking problems | tied to affected area (1) |
|  |  | palm | unknown | unknown (1) |
|  |  | head | unknown | unknown (1) |
| 2012 | 1 | bone | give strength | dried, ground, mixed with water, and used as enema for child (1) |
| **Dwarf Crocodile (***Osteolaemus tetraspis*) | | | | |
| 2017 | 2 | feces | swelling | ground and rubbed on affected area (2) |
| **Eagle** (*Accipitridae)* | | | | |
| 2017 | 2 | brain | mental problem | used to prepare medicine enhance intelligence (2) |
|  |  | eye | eye problem | used to prepare medicine to enhance eyesight (2) |
| **Elephant** (*Loxodonta cyclotis*) | | | | |
| 2017 | 11 | feces | swelling or bone problem | rub on swelling (2), rub on bone problem (1), or unknown ailments (1) |
|  |  | feces | wound | add to snuff (tobacco) to seal wound (1) |
|  |  | feces | dysentery | mixed with tree bark and local gin (distilled palm wine) and drank (1) |
|  |  | feces | epilepsy | mixed with herbs and rubbed all over the body (1) |
|  |  | bone | dislocation | bone is fixed to the same bone that is fractured in the patient's body (1) |
|  |  | skin | filariasis | dried skin is ground and rubbed on the body (1) |
|  |  | tusk | neck pain | tied around the neck (1) |
|  |  | limbs | walking problems (child) | wash the limbs of a animal with water, the resulting water is used to pump the stomach of a child (enema) (1) |
|  |  | tusk, teeth | unknown | sell to Hausa people (1) |
|  |  | synovial fluid (patella) | joint pains | mixed with palm oil and drunk by patients to cure joint pains (1) |
|  |  | synovial fluid (patella) | gonorrhea infection | mixed with bitter leaf and consumed (1) |
| 2012 | 7 | feces | wound | mixed with water and rubbed on wound (3) |
|  |  | feces | fever | mixed with local gin (distilled palm wine) and drank (1) |
|  |  | bile | stomach problem | mixed with local liquor and drank (1) |
|  |  | fat | cough | melted and drank (1) |
|  |  | stomach fluid | give strength | water from stomach drank (1) |
|  |  | stomach | give strength | stomach with water used for pillow (1) |
| **Flying Squirrel** (*Anomalurus beecrofti*) | | | | |
| 2017 | 11 | fur | burns or injury | rubbed/placed on affected area (10) |
|  |  | fur | catarrh cough | fur is grinded with herbs and consumed (1) |
| 2012 | 21 | fur | burn | rubbed on burn or other wound or sore (21) |
| **Galago** (*Galago* spp.) | | | | |
| 2017 | 1 | fur (tail) | burns | rubbed on affected area (1) |
| **Genet** (*Genetta genetta*) | | | | |
| 2017 | 2 | anus | toothache | unknown (1) |
| 2012 | 2 | skin | cough, cold | roasted then mixed with water and drank (2) |
| **Giant Pouched Rat** (*Cricetomys emini*) | | | | |
| 2017 | 2 | flesh | malaria | prepared and eaten as peppe soup (2) |
| 2012 | 4 | flesh | epilepsy | boiled and consumed (1) |
|  |  | gall bladder | gonorrhea | boiled and consumed (1) |
|  |  | intestine | swollen stomach | mixed with sweet yam and consume (1) |
|  |  | intestine | kidney problem | boiled with water consumed (1) |
| **Gorilla** (*Gorilla gorilla diehli*) | | | | |
| 2017 | 4 | bone | give strength | ground and mixed with water then consumed (1) |
|  |  | bone | give strength | mixed with herbs to perform a ritual (1) |
|  |  | bone | give strength | tied to child's neck and worn as they grow (1) |
|  |  | bone | give strength | ground bone and mix with water to pump baby (enema) (1) |
|  |  | flesh | strength for unborn child | consumed by pregnant women (1) |
| **Hornbill (***Bucerotidae*) | | | | |
| 2017 | 6 | head | unknown | sold to Hausa and Yoruba people who use for medicine (1) |
|  |  | beak | unknown | sold to Hausa and Yoruba people who use for medicine (1) |
|  |  | feathers | unknown | sold to Hausa and Yoruba people who use for medicine (1) |
|  |  | legs | unknown | unknown (3) |
| **Guinea fowl (***Guttera plumifera* or *Agelastes niger*) | | | | |
| 2017 | 2 | unknown | unknown | unknown (2) |
| **Leopard** (*Panthera pardus*) | | | | |
| 2017 | 3 | feces | joint pain | ground with other herbs, mixed with palm kernel oil, and rubbed on affected part (1) |
|  |  | skin | skin injury | unknown (1) |
|  |  | whiskers | epilepsy | put into water and consumed (1) |
| 2012 | 1 | flesh | cough | cooked and consumed (1) |
| **Monkey (***Cercopithecus* spp.) | | | | |
| 2012 | 62 | bone (skull) | cough, child's cough | used as cup to drink boiled water and herbs from (46) |
|  |  | feces | cough | drank with water/ local liquor (13) |
|  |  | intestine | cough | boiled and drank broth (2) |
|  |  | fur | prevent sickness | mixed with local liquor and drank (1) |
|  |  | bone (skull) | headache | ground, mixed with water and rubbed on head (1) |
| **Monitor lizard (***Varanus niloticus*) | | | | |
| 2017 | 10 | skin | cough | soaked in water and consumed daily (9) |
|  |  | fat | stomach pain | unknown (1) |
| 2012 | 7 | flesh | epidemic cough | boiled and broth drank (5) |
|  |  | skin | child's convulsions | dried in sun, used as lozenge (1) |
|  |  | skin | child's convulsions | dried in sun, wore on necklace (1) |
| **Palm civet** *(Nandinia binotata)* | | | | |
| 2017 | 1 | skin | skin injury | unknown - used as a replacement of leopard (1) |
| 2012 | 1 | feces | baby's stomach pain | mixed with water and used as enema for baby (1) |
| **Pangolin** (*Manis tricuspis* or *Manis tetradactyla*) | | | | |
| 2017 | 7 | head and fingers | stomach pain | placed on affected area (1) |
|  |  | skin | stomach pain | placed on affected area (1) |
|  |  | skin | unknown | sold to Yoruba people who use it for medicine (1) |
|  |  | scales | Jedi Jedi and spleen problems | burnt into ashes and ground, then added to palm oil for baby to consume (1) |
|  |  | scales | cough | roasted and eaten (1) |
| **Porcupine (***Atherurus africanus*) | | | | |
| 2017 | 1 | spines | asthma | roasted and the smell is inhaled (1) |
| 2012 | 7 | spines | pain, injury, or itchy black leg | burned and ashes used as rub (3) |
|  |  | spines | boil | laceration tool (1) |
|  |  | intestine | malaria, general sickness | boiled and drank as broth or consumed (2) |
|  |  | heart | stomach pain | mixed with alcohol and drank (1) |
| **Potto/ Angwantibo (***Perodicticus potto or Arctocebus calabarensis*) | | | | |
| 2017 | 9 | flesh (forelimb) | give fetus strength and ease delivery | cooked and dried and put in soup for pregnant woman to consume (5) (some put 6-inch nails into the pot to soften the tough meat) (some specify right forelimb as the useful part of body) |
|  |  | bone | weakness (baby) | roasted and ground, used to pump baby (i.e., as enema) (1) |
|  |  | bone | weakness (baby) | roasted and ground, used to mark baby (i.e., rubbed in cuts made with a razor blade) (1) |
|  |  | flesh | weakness of joints/ body (child) | meat is dried, ground into powder, and mixed with other herbs to rub into razor cuts made along the child’s chest, joints, and waist to cure weakness within 7 days (1) |
|  |  | flesh | weakness of joints/ body (child) | meat is dried, rubbed on limbs of child unable to walk (1) |
|  |  | hand and limbs | unknown | sold to Yoruba people for medicine (1) |
| 2012 | 5 | flesh (leg) | give strength to baby | flesh roasted and eaten by pregnant women (2) |
|  |  | flesh | cough, gonorrhea | boiled without ingredients and broth consumed (2) |
|  |  | flesh | hernia | cooked with leaves and consumed in large quantity (1) |
| **Puff Adder** (*Bitis arietans*) | | | | |
| 2017 | 1 | head | breast problems | rub on affected area (1) |
| **Python** (*Python sebae*) | | | | |
| 2017 | 31 | bile | poisoning/ stomach pain | added to local gin and one shot taken (person must wait 30 minutes before drinking water or else will die) (9) |
|  |  | fat | inflammation/ rheumatism | apply to anything inflamed (8) |
|  |  | fat | dislocation/ bone problem/ body pain | melted and oil is applied to the affected area (8) |
|  |  | fat | cough | sundried and sucked on as lozenge (1) |
|  |  | fat | injury | applied to affected area after cutting it with razor blade (1) |
|  |  | teeth | lactation problem | used to make a cut on breast to relieve pain (1) |
|  |  | teeth | snake bite treatment | used to make a cut where snake bite occurred to relieve pain (1) |
|  |  | bile | epilepsy | mixed with other ingredients and taken orally (1) |
| 2012 | 35 | fat | dislocation/ body pain | used as rub on affected area (14) |
|  |  | fat | fever/ malaria | mixed with liquor and consumed (4) |
|  |  | fat | cough | dried in sun to use as lozenge (1) |
|  |  | bile | poisoning | put in local liquor, consume, wait 30 minutes before drinking water to avoid death (8) |
|  |  | kidney | malaria, body pain | put in local liquor and drank (3) |
|  |  | teeth | breast pain | used as a laceration tool to release pain of breast (3) |
|  |  | teeth | boil | used as a laceration tool to release pain of boil (1) |
|  |  | flesh | body pain | cooked and consumed (1) |
| **Red duiker (***Cephalophus dorsalis* or *Cephalophus ogilbyi*) | | | | |
| 2017 | 15 | flesh (tail) | eases childbirth, prevents miscarriage | cooked and mixed with herbs and given to pregnant women to help expand the cervix or who have previously suffered a miscarriage (3) |
|  |  | flesh (leg) | eases childbirth | dried leg is roasted and dipped into a cup containing water and the resulting extract is given to a pregnant woman (1) |
|  |  | bone | rib pain | bone is ground and rubbed in skin that was cut with razor (1) |
|  |  | flesh (thigh) | severe headache, migraine, epilepsy | dried meat ground, mixed with herbs, and applied in razor blade marks made on the forehead (4) |
|  |  | flesh | enhances intelligence in children | cooked and eaten with other herbs (1) |
|  |  | flesh (neck) | heart disease | cooked and eaten with other herbs (1) |
|  |  | flesh (neck) | fibroids | cooked and eaten with herbs (1) |
|  |  | pancreas | pain | unknown (1) |
| 2012 | 5 | skin | epilepsy, malaria | roasted, mixed with water or alcohol, and drank (4) |
|  |  | flesh (thigh) | malaria | mixed with herbs and consumed (1) |
| **Red river hog (***Potamochoerus porcus*) | | | | |
| 2017 | 2 | ear | swollen jaw (child) | cooked and eaten (1) |
|  |  | unknown | unknown | unknown |
| 2012 | 14 | bone | boil | mixed with water and rubbed on boil (14) |
| **Snail** (*Achatina achatina*) | | | | |
| 2017 | 3 | shell | eye infection | ground and rubbed on eye (1) |
|  |  | shell | walking problem (child) | shell used to store medicine that is applied to an area of the leg after cutting with razor blade (1) |
| 2012 | 1 | flesh | spit in eye from cobra | rubbed in eye after a snake spits in it (1) |
| **Tortoise** (*Kinixya erosa*) | | | | |
| 2017 | 19 | feces | rheumatism | collected, dried, mixed with alligator pepper until smooth, and applied on the portion affected after it has been pierced and little razor cuts have been made (1) |
|  |  | shell | convulsions, epilepsy | burnt and blended with pepper and rubbed on body/chest or mixed with palm oil and consumed (2) |
|  |  | shell | cough, tuberculosis, breathing difficulty | burnt and blended with pepper and rubbed on body/chest (1) |
|  |  | shell | cough, tuberculosis, breathing difficulty | mixed with palm oil and consumed (1) |
|  |  | brain | brain function | dried, ground, mixed with native leaves and used as rub (1) |
|  |  | brain | brain function | mixed with native leaves and consumed as peppe soup (2) |
|  |  | bone | bone pain, sprain, or dislocation | tied around affected area (2) |
|  |  | shell | unknown | ground and rubbed on body (1) |
|  |  | flesh (leg) | unknown | cooked and consumed (1) |
|  |  | head | unknown | sold to Yoruba people for unknown medicine (1) |
|  |  | shell | stomach pain | stores medicine that is later applied to and cut made from razor blade on stomach (1) |
|  |  | shell | unknown | usage only known by the witch doctor (1) |
|  |  | skin | stomach pain | boil and rub on affected area (1) |
| 2012 | 12 | bone | dislocation, waist pain, joint pain | tied to body where affected (5) |
|  |  | bone or shell | give child strength | ground, mixed with water, and used as enema (2) |
|  |  | bone or shell | give child strength | ground, rubbed in scarifications (2) |
|  |  | bone or shell | give child strength | ground, given to child with food (1) |
|  |  | intestine | tuberculosis, cough | mixed with palm oil and drugs, drank (1) |
|  |  | heart | tuberculosis, cough | mixed with palm oil and drugs, drank (1) |
|  |  | skin | tuberculosis, cough | mixed with palm oil and drugs, drank (1) |
| **Viper** (*Viperidae*) | | | | |
| 2012 | 1 | teeth | breast cancer | used to bleed breast (1) |
| **Water chevrotain** (*Hyemoschus aquaticus*) | | | | |
| 2017 | 9 | flesh/ skin/ bone (leg) | epilepsy, convulsions, high fever, severe cold | roasted and soaked in water for days and water is taken twice a day (7) |
|  |  | skin | fever | roasted and the smoke is inhaled while roasted (1) |
| 2012 | 42 | flesh/ skin/ bone (leg) | fever chills | roasted, ash mixed with water or local liquor and drank (41) |
|  |  | bone | promote child's growth | roasted, ash mixed with water and used as an enema (1) |
| **Turaco (***Corythaeola cristata*) | | | | |
| 2017 | 4 | feathers | whooping cough | feathers from the tail are roasted and ash is mixed with palm kernel oil and taken orally (1) |
|  |  | bone marrow | whooping cough | animal is cooked and the bone marrow is sucked (1) |
|  |  | head | unknown | sold to Yoruba people for medicine (1) |
|  |  | feathers | unknown | sold to Yoruba people for medicine (1) |
|  |  | leg | unknown | sold to Yoruba people for medicine (1) |

*^1^ Indicates the number of times an animal was listed by participants in each study year.*

*^2^ Numbers listed indicate the number of times participants described a medicinal use.*

**Table S3.** Cultural practices involving animals.

| **Year** | **#** ^1^ | **Body part** | **Treatment** | **Preparation and administration**^2^ |
| --- | --- | --- | --- | --- |
| **African civet** (*Civettictis civetta*) | | | | |
| 2017 | 1 | skin | display | dried skin is worn by masquerade dancing group (1) |
|  |  | skin | display | dried skin is used to make drums for masquerade dancing group (1) |
| **Blue duiker** (*Philantomba monticola*) | | | | |
| 2017 | 14 | flesh | ceremonial consumption | consumed during marriage, burial, birthdays, etc. (5) |
|  |  | skin | display | skin is dried and used to make traditional drums (3) |
|  |  | flesh | gift | hunters donate meat during New Yam Festival, where it is cooked and consumed by the entire community (1) |
|  |  | skin | charm | skin dried and used in African bullet proof charm (1) |
| 2012 | 33 | flesh | sacrifice | sacrificed to ancestors during Akarikpo festival to provide fertility to the land (29), to appease any problem with the gods (1) |
|  |  | flesh | ceremonial consumption | consumed during any festival (1), consumed during New Yam festival (1) |
|  |  | flesh (thigh) | gift | given to elders for consumption during New Yam festival (1) |
| **Boa** (*Calabaria reinhardtii*) | | | | |
| 2017 | 1 | skin | display | used as decoration for chief's house (1) |
|  |  | skin | display | dried and used to make hats |
| **Buffalo** (*Syncerus caffer*) | | | | |
| 2017 | 5 | horn | display | used as a traditional trumpet after being skillfully perforated, then blown during traditional dances (1), or as a community alarm during emergencies or to find someone lost in the forest (1) |
|  |  | head and body parts | gift | the head is reserved for the hunters age grade, one part of the body is given to the community (chiefs), and the rest is kept by the hunter (1) |
|  |  | flesh | sacrifice | sacrificed when a great man dies (1) |
|  |  | feces | charm | mixed with goat skin, herbs, and tied around the waist of a warrior as a bullet proof charm (1) |
| **Cat** (*Felis catus*) | | | | |
| 2017 | 1 | skin | display | used for drum (1) |
|  |  | skin | display | for decoration for chief's house (1) |
|  |  | skin | display | used to make hats (1) |
| **Chicken** (*Gallus gallus*) | | | | |
| 2017 | 17 | whole animal and blood | sacrifice | live animal is killed, and blood is sprinkled on ground to cleanse and appease gods after a crime (11) |
|  |  | flesh, tail, gizzard | ceremonial consumption | used to entertain important visitors and/or family during Christmas period and other festivities (5), tail and gizzard are particularly important (1) |
|  |  | whole animal, egg | gift | whole animal (1) or egg (1) demanded by a Juju doctor before he makes medicine |
|  |  | live animal | display | during ceremonies, the leader of a masquerade may hold a live chicken or use it to fan the chief (2) |
| **Cow** (*Bos taurus*) | | | | |
| 2017 | 4 | thigh, heart, kidney | gift | reserved for title holders and elders as a sign of respect (1) |
|  |  | tail | display | during a Masquerade dance the tail is thrown and one must throw it back and pay (1) |
|  |  | flesh | ceremonial consumption | killed and consumed during ceremonies (1) |
|  |  | whole animal | fine | payment to the chiefs if a crime is committed (1) |
| **Dog** (*Canis lupis*) | | | | |
| 2017 | 3 | flesh | ceremonial consumption | used by a group that values the dog (Emberembit): members eat the dog; dog is offered whenever a group member dies (1) (Ayo culture). When a member of the community is perceived to have been struck dead by [Ekperokwa] deity, a dog is killed by Ekperokwa initiates and used in a cleansing ritual for Ekperokwa shrine (1) (Ejagham culture). |
|  |  | live animal | sacrifice | unknown sacrificial use (1) |
|  |  | blood | sacrifice | used by a group that values the dog (Emberembit): new initiates must present dog as gift to gods, dog is used during dancing ceremony, where members kill a dog, transfer blood into human skull |
| **Drill Monkey (***Mandrillus leucophaeus*) | | | | |
| 2017 | 3 | flesh | ceremonial consumption | consumed for any ceremony or festival (2) |
|  |  | skin (with fur) | display | used for male initiation where the mane is tied on the right hand of the male and the left hand of the female during a dance (1); held while playing (dancing) Ekpe (1) |
|  |  | unknown | charm | used to do unknown magic (1) |
| **Dwarf Crocodile** (*Osteolaemus tetraspis*) | | | | |
| 2017 | 6 | head | charm | the head is used to make a signal to (Angbu) deity (Ejagham culture) (1) |
|  |  | whole animal | display | animal is displayed during Ekpe meeting, some incarnation is performed to bring out Dwarf Crocodile life (1) |
|  |  | skin | charm | the back skin is dried and worn as a bullet proof war charm during war (1) |
|  |  | skin | display | the skin is removed, dried, and used for drums (1) |
|  |  | scales | charm | scraped off animal and mixed with other [unspecified] ingredients to use for protection or protect farms from theft (1) |
|  |  | flesh | sacrifice | unknown (1) |
|  |  | flesh | gift | when killed, part of the animal is given to the chiefs in the community (1) |
| **Eagle** *(Accipitridae)* | | | | |
| 2017 | 1 | feathers | display | feathers used to decorate chiefs’ hat and signify peace (eagle) |
| **Elephant (***Loxodonta cyclotis*) | | | | |
| 2017 | 12 | tail | display | tail used for dance costume and held in the hands of the dancers (Moni-nkim) and community leaders (1) |
|  |  | tusk/teeth | display | tusk and teeth used for decoration of chief's palace and as royal regalia for the highest chief (4) |
|  |  | tusk | display | tusk is blown as a trumpet during special occasions (2) |
|  |  | tusk | display | displayed during cult activities (1) |
|  |  | whole animal | gift | when killed, the tail is given to the highest profile chief to signify his authority and fame (1); the upper part is reserved for the hunter, whereas the underneath is reserved for the community, and head is given to the head of the household (1); part of the trunk, heart, tusk, and hind/fore limb are presented to the community (2) |
|  |  | tusk | charm | incantations are said before gifting the tusk to the highest chief, making any command given by him valid and undisputable (1) |
| 2012 | 1 | flesh | sacrifice | sacrificed in celebration of New Yam festival in the past (1) |
| **Flying squirrel** (*Anomalurus* *beecrofti*) | | | | |
| 2017 | 1 | skin | charm | skin is used to make, or repair charms used during war (for disappearance) (1) |
| **Genet** (*Genetta genetta*) | | | | |
| 2017 | 4 | skin (with tail) | display | dried skin and tail are displayed during dancing groups (Moni-nkim) (3) |
|  |  | skin | display | used for palace decoration (1) |
| **Goat** (*Capra aegagrus*) | | | | |
| 2017 | 32 | flesh | ceremonial consumption | goat provides entertainment for many celebrations, including chief’s coronation, New Yam festival, Christmas, and burials and masquerade dances, where it is typically shared to chiefs, women, and youth groups for eating (11) |
|  |  | blood | sacrifice | sacrificed for Juju doctor, Ekpe (leopard society) initiates, during chieftain coronation, during New Yam Festival, and/ or to appease community gods after a law is broken by cleansing ground with blood (10) |
|  |  | whole animal | fine | used as a fine and/or brought to a chief to settle a dispute (8) |
|  |  | skin with fur | display | skin/fur removed and given to chief to dry it and use to decorate house (2); |
|  |  | tail | display | held during dancing (2)/ tail is thrown at people who have to throw it back and pay during a masquerade dance (3) |
| **Guinea fowl (***Guttera niger*) | | | | |
| 2017 | 3 | flesh | sacrifice | Juju sacrifice (1) |
|  |  | feathers | display | decorate the hair of female dancers (Moni-nkim dance) and chief’s hats (2) |
| **Hornbill (***Bucerotidae*) | | | | |
| 2017 | 1 | head and legs | charm | used by native doctors to prepare African aircraft (translocation) charm (1) |
| **Leopard (***Panthera pardus*) | | | | |
| 2017 | 14 | skin | display | worn as traditional regalia by high members of the leopard (Ekpe) society and used to decorate the Chief's chair, palace, and Ekpe meeting hall to signify strength and authority (10) (women initiates are prevented from seeing the leopards face and anybody who is not an initiate of Ekpe cannot witness how the dissection process is done) |
|  |  | skin | display | displayed by hanging leopard around hunters’ neck who is given special status for killing it (4) |
|  |  | flesh | gift | when a leopard is killed the meat is given to members of the leopard society (Ekpe) only (1) |
| 2012 | 1 | whiskers | poison | put in water to poison someone who drinks it (1) |
| **Monkey** (*Cercopithecus spp.*) | | | | |
| 2017 | 3 | whole animal | display | Mona monkey (*Cercopithecus mona*) is used as a totem in Ekpe dance |
|  |  | skin | display | dried skin of any monkey is worn by the leaders of masquerade group |
|  |  | skin | display | dried skin is used to make drums |
|  |  | hands | charm | traditional drum beaters use the hands of the monkey to prepare a rub for their hands to improve drum beating (1) |
| **Palm civet** (*Nandinia binotata*) | | | | |
| 2017 | 3 | skin with fur | display | decorates attire for Moni-nkim dance (1) |
|  |  | skin with fur | display | interior decoration (1) |
|  |  | skin | display | making drums (1) |
|  |  | flesh | ceremonial consumption | used in preparing special meal during burial ceremony for chief (2) |
| **Pangolin** (*Manis* spp.) | | | | |
| 2017 | 1 | unknown | charm | war charm known by a different tribe (1) |
| **Parrot** *(Psittaciformes)* | | | | |
| 2017 | 1 | feathers | display | feathers used to decorate chiefs’ hat and signify protection (parrot) |
| **Porcupine** (*Atherurus africanus*) | | | | |
| 2017 | 9 | flesh | ceremonial consumption | used to celebrate any occasion in the community, including during age grade meeting or to unveil New Yam festival (dried meat is cut into pieces and shared by chiefs and others present) (6) |
|  |  | whole animal | gift | brought to the elders of the community to bring good luck (1)/fortune to community; presented to age grade by a stranger who wants to join (1) |
|  |  | spines | tool | used to braid women’s hair (1) |
|  |  | flesh | fine | in the absence of a goat, porcupine can be used as a sign of a fine settlement for any offender |
| 2012 | 1 | flesh | ceremonial consumption | used to celebrate any festival (1) |
| **Potto/ Angwantibo** (*Perodicticus potto or Arctocebus calabarensis*) | | | | |
| 2017 | 1 | hands and legs | charm | used in preparing African bullet proof charm (1) |
| **Python** (*Python sebae*) | | | | |
| 2017 | 7 | bile | gift | when killed, the head, part of the trunk, the tail, and the bile are brought to the chief palace by the hunter who killed it/ bile (which is considered poisonous) must be given to the community where it is destroyed in front of a delegation to ensure it is not used to bring harm (7) |
|  |  | skin | display | decoration of dance attire and chief’s palace (1) |
|  |  | skin | display | used to make drums (1) |
|  |  | whole animal | charm | people can transform into python (1) |
|  |  | flesh | charm | python put in local gin and consumed to make transformation into a python ineffective (1) |
|  |  | head | charm | unknown use by the Ancient and Mystical Order Rosae Crusis (AMORC) to cure spiritual attack (1) |
| **Red duiker (***Cephalophus dorsalis/ ogilbyi*) | | | | |
| 2017 | 27 | flesh | ceremonial consumption | eaten during any occasion, including marriage, circumcision, New Yam, and burial (7) |
|  |  | whole animal | gift | presented by strangers who want to become indigens (2), or hunt and/or collect non-timber forest products in community forests (2)/ given to women’s groups by woman with first time pregnancy (1)/ man must present red deer when he impregnates woman to show he is responsible for the pregnancy (1)/ given to chiefs as notification of burial (1) |
|  |  | flesh | sacrifice | used to appease gods of procreation (1)/ withhold village laws (Anugbu diety)/ during New Yam festival (1) |
|  |  | flesh (thigh) | fine | for any offense committed in the community (3) |
|  |  | flesh (thigh) | gift | must be given to the chiefs whenever this animal is killed (2) |
|  |  | flesh (thigh) | charm | used in ritual before the New Yam festival can commence and prior to burial in community (2) |
|  |  | skin | charm | cut into small pieces and spread in the farm to increased crop yield (1) |
|  |  | skin | charm | used in preparation of bullet proof charm (1) |
|  |  | skin | display | used for making drums (1) |
| 2012 | 7 | flesh | ceremonial consumption | used to celebrate any festival, including Christmas, New Yam, or marriage |
| **Red river hog (***Potamochoerus porcus*) | | | | |
| 2017 | 11 | head | gift | head is given to chiefs and age grade, and after eating the head, the age grade member donates money to re-empower the hunter (2) |
|  |  | flesh (thigh) | gift | intending member of Ekpe society must offer the thigh (1) |
|  |  | flesh | gift | given to community (chief) when killed, and more if killed by an stranger living in the community (4) |
|  |  | flesh (thigh) | ceremonial consumption | used for any occasion (1)/ youth chiefs and women share and eat during (Moni-nkim) dance (1) |
|  |  | bone (skull) | display | used as decoration in chief's palace (1) |
|  |  | flesh (thigh) | fine | for any crime committed in the community (1) |
| 2012 | 1 | flesh | ceremonial consumption | hunted and consumed to celebrate New Yam (1) |
| **Sitatunga (***Tragelaphus spekei*) | | | | |
| 2017 | 3 | skin | display | dried skin is used for making traditional drums (1) |
|  |  | skin | display | dried skin is used as foot mat for chiefs (1) |
|  |  | head and thigh | gift | when killed, the head is reserved for the hunter's age grade and thigh is reserved for the community (chiefs) (1) |
| **Tortoise (***Kinixya erosa*) | | | | |
| 2017 | 11 | shell | display | empty shell is used as a gong to call initiative for meeting and to pass information of public importance (2) or a musical instrument (1) |
|  |  | shell | display | play (dance) Ekpe (1) |
|  |  | shell | display | decoration of shrine (2) |
|  |  | flesh | sacrifice | used as small juju because it is a small animal (2)/ to appease gods in case of misbehavior (1) |
|  |  | shell | charm | used to prepare African bullet proof charm (1) |
|  |  | shell | charm | used to protect farms from theft (1) |
|  |  | scales | charms | scraped off and mixed with other ingredients to prepare charms used for protection (1) |
| 2012 |  | flesh | sacrifice | sacrificed to keep leprosy away (1) |
| **Turaco** (*Corythaeola cristata*) | | | | |
| 2017 | 1 | feathers | display | feathers used to decorate chiefs’ hat and signify strength (turaco) |
| **Water chevrotain (***Hyemoschus aquaticus*) | | | | |
| 2017 | 4 | head and thigh | gift | when killed, the head is reserved for the hunter's age grade, and the thigh is reserved for the community (chiefs) (2) |
|  |  | skin | display | skin is worn to display the character of a water chevrotain (1) |
|  |  | skin | display | used to make drums (1) |
| 2012 | 1 | flesh | sacrifice | protection of the village (1) |
| **Yellow-backed duiker (***Cephalophus sivicultor*) | | | | |
| 2017 | 1 | tail | display | used for dance (1) |
|  |  | whole animal | charm | people can transform into yellow backed duiker to show how strong they are or to destroy farms of their enemies (1) |
| 2012 | 1 | flesh | sacrifice | sacrifice before shrine during New Yam festival |
| **Any animal** | | | | |
| 2012 | 275 | flesh | ceremonial consumption | hunted and consumed during New Yam festival (village elders give order all men to go find bushmeat two weeks prior to festival) (202); also used to celebrate Christmas (33); New year (14); Easter (10), or any other festivities (e.g., dances, welcome visitors, marriage, burial, children's day, or other festival (14)) |
|  |  | flesh | sacrifice | given to ancestors to improve land (2) |

*^1^ Indicates the number of times an animal was listed by participants in each study year.*

*^2^ Numbers listed under preparation and application indicate the number of times participants described a cultural practice.*
